# Molecular Mechanism of Ciprofloxacin Translocation Through the Major Diffusion Channels of the ESKAPE Pathogens *Klebsiella pneumoniae* and *Enterobacter cloacae*

**DOI:** 10.1021/acs.jpcb.4c03327

**Published:** 2024-08-24

**Authors:** Abhishek Acharya, Pratik Kumar Behera, Ulrich Kleinekathöfer

**Affiliations:** School of Sciences, Constructor University, Campus Ring 1, 28759 Bremen, Germany

## Abstract

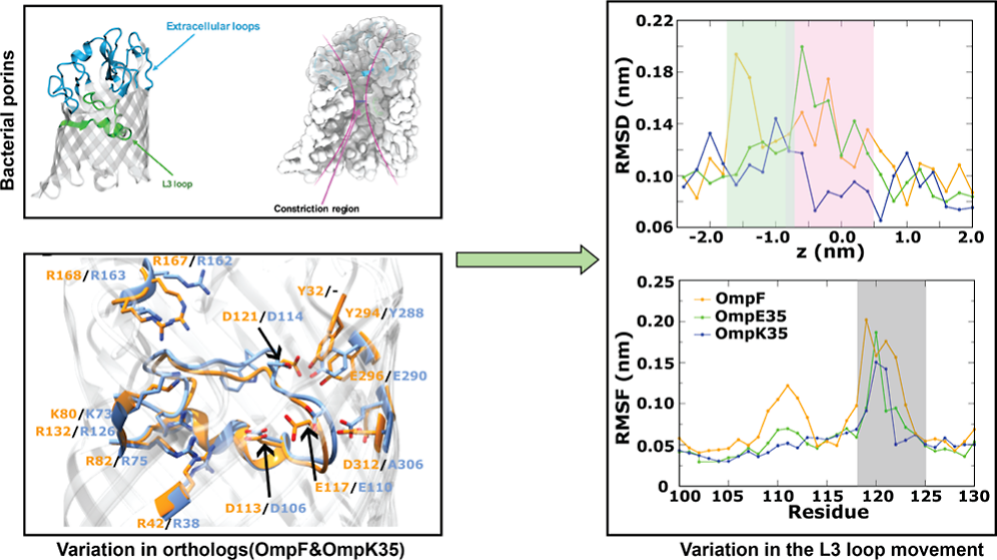

Experimental studies on the translocation and accumulation of antibiotics in Gram-negative bacteria have revealed details of the properties that allow efficient permeation through bacterial outer membrane porins. Among the major outer membrane diffusion channels, OmpF has been extensively studied to understand the antibiotic translocation process. In a few cases, this knowledge has also helped to improve the efficacy of existing antibacterial molecules. However, the extension of these strategies to enhance the efficacy of other existing and novel drugs require comprehensive molecular insight into the permeation process and an understanding of how antibiotic and channel properties influence the effective permeation rates. Previous studies have investigated how differences in antibiotic charge distribution can influence the observed permeation pathways through the OmpF channel, and have shown that the dynamics of the L3 loop can play a dominant role in the permeation process. Here, we perform all-atom simulations of the OmpF orthologs, OmpE35 from *Enterobacter cloacae* and OmpK35 from *Klebsiella pneumoniae*. Unbiased simulations of the porins and biased simulations of the ciprofloxacin permeation processes through these channels provide insight into the differences in the permeation pathway and energetics. In addition, we show that similar to the OmpF channel, antibiotic-induced dynamics of the L3 loop are also operative in the orthologs. However, the sequence and structural differences, influence the extent of the L3 loop fluctuations with OmpK35 showing greater stability in unbiased runs and subdued fluctuations in simulations with ciprofloxacin.

## Introduction

The increasing global prevalence of antimicrobial resistance poses a significant risk of future epidemics in human populations. At the same time, prevention and treatment of common bacterial infections are becoming less effective against strains that have developed multidrug resistance and, in some cases, extreme drug resistance.^1^ The World Health Organization has emphasized the urgent need to address the emergence of resistance in clinical pathogens, most prominently the ESKAPE pathogens, that are the leading cause of resistance-associated deaths.^1^ Resistance in these species significantly increases the morbidity and mortality associated with nosocomial infections. The recent Global Antimicrobial Resistance and Use Surveillance System (GLASS) report draws attention to the worrying increase in resistance rates among bacterial pathogens, such as a 42% rate of *Escherichia coli* resistant and a resistance rate of more than 59% of *Klebsiella pneumoniae* to third-generation cephalosporins.^2^

The development of new drugs against resistant pathogens faces significant hurdles not only in identifying candidates that demonstrate effectiveness during *in vitro* assessment but also in ensuring their *in vivo* efficacy and safety. Identification of drug candidates that are effective in *in vitro* studies on pathogenic isolates is a primary challenge. Low accumulation of drug molecules inside the bacterial cell has been identified as a reason for the failure at this stage. Gram-negative bacteria in particular are difficult targets due to the outer membrane with a dense layer of lipopolysaccharides that is impermeable to most polar solutes.^3−6^ The influx of polar solutes, including antibiotics, takes place *via* the general diffusion channels, also called porins, that are embedded within the outer membrane.^7−15^ At the same time, a process that counteracts the accumulation of drug molecules within bacterial cells, *i.e.*, efflux, is active and is attributed to the action of ATP-driven efflux plumps. The net accumulation is therefore largely determined by the influx and efflux rates across the bacterial membrane. Naturally, in drug development efforts, strategies have been considered to improve the influx and reduce the efflux rates for existing or novel drug molecules.

**Figure 1:**
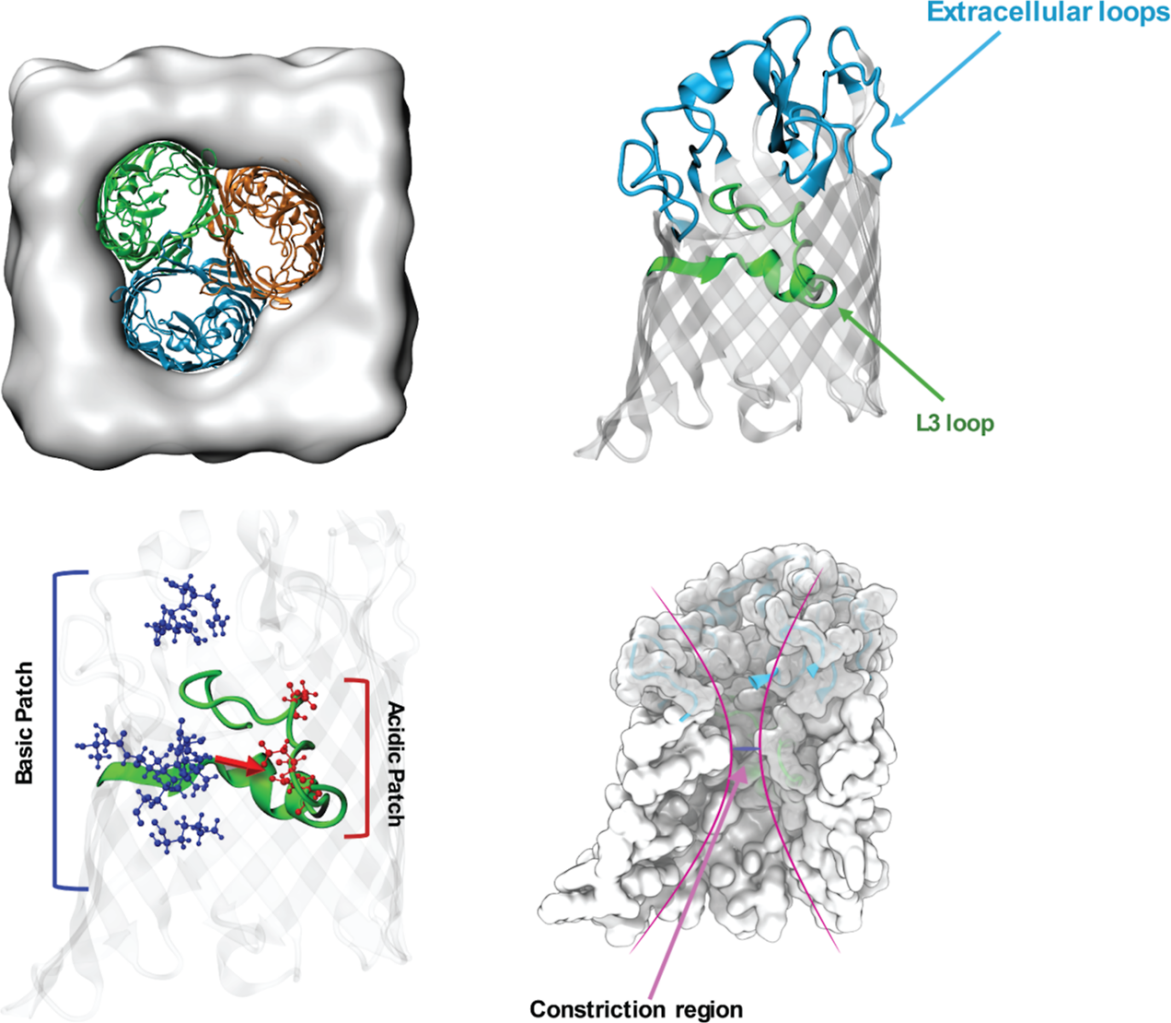
General structural features common to OmpF and its orthologs. These porins are composed of 16-stranded β-barrels arranged in the form of a trimer. The β-strands are connected *via* long loops toward the extracellular side of the barrel. The L3 loop is folded back into the lumen of the barrel, partially occluding the channel and leading to an hourglass shape with a CR at the center of the pore. The CR is also characterized by the presence of a strong transverse electric field that arises due to the presence of charged residues of opposite polarity.

Mechanistic insights into the antibiotic permeation processes through porins are expected to aid in the development of drugs with improved influx properties. The porin OmpF and its orthologs are of particular interest due to experimental evidence of their role in the influx of drugs from different antibiotic classes such as carbapenems, fluoroquinolones and penicillins.^6,16−21^ Porins usually exist as trimers, wherein each monomer is a β-barrel formed by antiparallel β-sheets (see Figure 1). The external hydrophobic surfaces serve as intermonomer contacts, along with an extracellular loop L2 that forms polar interactions with a groove of the neighboring monomers. Several long loops form the extracellular opening of the channel, except for the loop L3 that folds inward into the channel lumen to create a narrow constriction region (CR) which is responsible for the size exclusion property of the channel. In the case of *E. coli*, the porins OmpF and OmpC have almost circular constriction zones with a diameter of approximately 6.5–7 and 5.5–6 Å, respectively. The CR is characterized by a transverse electric field generated due to the presence of positively and negatively charged residues that decorate the opposite sides of the CR (Figure 1).^16,22^ In a previous study on OmpF and its homologues,^23^ a transversal field of around 0.15 to 0.30 V/nm in the CR was reported. This transverse electric field in the CR has been shown to play a critical role in the permeation of polar solutes through the pore.^9,23^ It aids in orienting solutes with an internal dipole into configurations that maximize the engagement with the charged residues in the CR. This strong orientation and the interactions with the residues in the CR help the solute molecule somewhat offset the potential barrier that arises due to the steric restriction in accommodating bulky solutes. Mutations of the charged residues in the CR have been implicated in the development of resistance.^22^ Experimental investigations combined with molecular simulations have also identified the other molecular determinants of permeation that include antibiotic properties such as size, electrostatics (net charge and distribution) and flexibility as well as channel properties including pore electrostatics, radius, and loop dynamics.^18,21,23−31^ These factors were also considered in the development of a quantitative scoring function that can be used to predict the permeability of a given channel for a set of antibiotics.^23^ Additionally, empirical studies have demonstrated the importance of a positively charged moiety in the accumulation of drugs inside bacterial cells and the findings were successfully applied toward the development of antibiotic molecules with improved efficacy and broader antibacterial spectrum.^32−35^ Detailed simulations of the OmpF channel have uncovered the mechanistic basis for the preference of molecules with a positive charge and the role of antibiotic-induced loop dynamics in the translocation of antibiotics.^36^ Using simulations on the permeation of antibiotics with different charges, the study showed that an accessible positively charged moiety can interact with acidic residues on the L3 loop and therefore can potentially induce transient conformational shifts in the flexible L3-FS segment (F118–S125) of the loop during permeation through the narrow CR. This mechanism has been termed L3-dynamics dependent (L3D-D) translocation. In contrast, molecules with only negative charged moieties prefer to interact with the basic residues of the barrel wall and do not induce significant conformational fluctuations in the L3 loop resulting in an L3-dynamics independent (L3D-I) translocation mechanism. The corresponding permeation model provides a possible explanation for the findings from previous empirical investigations regarding the importance of a sterically accessible positively charged group and is also consistent with reports of a fast permeation of zwitterionic antibiotics.^7,16,23,32,33^ While these studies have focused on the OmpF channel as a model, the implied role of the L3 loop dynamics in antibiotic translocation necessitates an examination of a similar role of the loop dynamics in the porin orthologs from other pathogens of clinical significance. Moreover, notable structural variations among the orthologs have been suggested to govern the experimentally observed differences in the permeation rates of a given antibiotic through the orthologs. A detailed atomistic description of the antibiotic permeation process through the OmpF orthologs and comparisons with OmpF may enable to clarify the effect of the detailed pore structure and dynamics on the exact permeation pathway.

With this objective, the present work focuses on the orthologs of OmpF, namely OmpE35 from *Enterobacter cloacae* and OmpK35 from *K. pneumoniae*. To this end, we begin with the analysis of the available crystal structures^23^ to examine the pore structure and amino acid variations in and around the CR of the orthologs that may influence the pore dynamics and antibiotic permeation, providing a structural basis for the expected differences in dynamics of the L3 loop. Unbiased MD simulations of the channel have been performed to examine the intrinsic L3 loop stability in the absence of an antibiotic. This part is followed by detailed simulations of ciprofloxacin (CIP) permeation through the orthologs. CIP was chosen as the molecule of interest because it is a rigid, zwitterionic molecule, and its permeation pathways through OmpF are associated with significant conformational fluctuations of the L3 loop.^31^ Since the permeation process is essentially a rare event in the practically accessible simulation time scales, we employed an enhanced sampling scheme to investigate the permeation process. Our approach employs the temperature accelerated sliced sampling^37^ (TASS) method which is similar to the umbrella sampling method that uses a series of harmonic bias potentials for sampling antibiotic configurations along the channel axis, but also enables within each simulation window an improved sampling of the antibiotic translation and rotational degrees of freedom by boosting the sampling along the associated collective variables (CVs). The method has been previously used to calculate free energy for the permeation of different antibiotics through OmpF and provided qualitative insights into antibiotic induced L3 dynamics.^31,36,38^ Structural analyses and simulations with antibiotics indicate that the observed sequence variation between the homologues determine the stability and (antibiotic-induced) dynamics of the L3 loop.

## Materials and Methods

### System Setup

The simulation systems were prepared using the atomic coordinates of OmpF (*E. coli*) (PDB ID: 2ZFG) and its orthologs OmpK35 (*K. pneumoniae*) (PDB ID: 5O77), and OmpE35 (*E. cloacae*) (PDB ID: 6ENE) obtained from the Protein Data Bank. The trimeric forms of these channels were embedded into a lipid bilayer with the help of the Membrane Builder module within the CHARMM-GUI server.^39,40^ All titrable residues were modeled in their standard protonation states at pH 7.0 except residues E296 in OmpF, D285 in OmpE35 and residues E102 and E110 in OmpK35 (see Supporting Information Section S1 for details). The lipid bilayer consists of 1-palmitoyl-2 oleoyl-*sn*-glycero-3-phosphoethanolamine (POPE) molecules. TIP3 water molecules were used to solvate the protein–membrane system, and neutralization was achieved by adding potassium ions. For the unbiased simulations, additional K^+^ and Cl^–^ were added to obtain a net concentration of 0.15 M. The details of these systems are provided in Table S1. The systems were simulated using the CHARMM36 force field^41,42^ with the short-range electrostatics and the van der Waals interactions calculated using a cutoff of 12 Å and a switching distance of 10 Å. The long-range electrostatics was treated using the particle-mesh Ewald approach^43^ with a grid spacing of 1 Å. Moreover, all bonds were constrained using the parallel LINCS algorithm.^44^ A minimization step was performed using the steepest-descent algorithm, and equilibration was performed in steps for a total of 50 ns. Final production runs were performed in the *NPT* ensemble. The temperature was set at 300 K using the Nose–Hoover thermostat with a 1 ps coupling constant, and the pressure was maintained at 1 bar using a semi-isotropic scheme with the Parrinello–Rahman barostat. The unbiased production simulations were performed for a total of 150 ns. For the biased simulations, we used a virtual site setup^45−47^ that enabled utilizing a 5 fs time step. For setting up the virtual site systems, the pre-equilibrated all-atom system topology was converted to the virtual topology using the pdb2gmx tool in GROMACS. Thereafter, an equilibration step was performed with position restraints initially applied on all heavy atoms of the protein and antibiotic molecule as well as the phosphate atoms of the lipids. The equilibration was performed with a gradual release of the restraints in a stepwise manner. Simultaneously, the time step was increased from 1 to 2 fs and finally to 5 fs in the final equilibration step, as described in a previous study.^48^ All simulations were performed with GROMACS 2019^49^ patched with PLUMED plugin version 2.4.^50^ The force field parameters for the CIP molecule were obtained from a previous study.^48^ UCSF Chimera,^51^ VMD^52^ and in-house Python scripts were used to analyze the trajectory data and to create images for this work.

### Choice of Collective Variables

The biasing strategy used in this study enables accelerated sampling along multiple CVs. The principle CV *z* is defined as the projection of the vector between the center of mass of the antibiotic molecule and the C_α_ atoms of the β-strands of the channel along the *z*-axis. The CV approximates the direction of permeation through the channel and has been used in previous studies.^38,53,54^ In addition, CVs describing the rotation and translation of the antibiotic, and antibiotic–solvent interactions were included in the sampling scheme. The translational CVs *x* and *y* describe the motion of the antibiotic molecule orthogonal to the pore axis in *x* and *y*-direction. Mathematically, these are the projections of the vector between the center of mass of the antibiotic and the C_α_ atoms of the channel along the *x* and *y*-axes, respectively (Figure S2A). The rigid body rotation of the antibiotic molecule is described through additional CVs. The CVs *z*_*ij*_ and *x*_*ij*_ denote the projections of the internal antibiotic vector *r*_*ij*_ between the carbonyl carbon atom (C16) and the nitrogen atom of the piperazine ring along the *z* and *x*-axes, respectively. Moreover, *y*_*kl*_ and *z*_*kl*_ describe the projections of the internal antibiotic vector *r*_*kl*_ between the C2 and C4 atoms of the quinolone ring onto the axes *y* and *z*, respectively. The two internal vectors are shown in Figure S2B. The rotation of the antibiotic along the specified axis is defined as θ = cos^–1^(*p*_*ab*_/∥***r***_*ab*_∥), where *p*_*ab*_ denotes the respective projection of the vector ***r***_*ab*_ along the given axis. In practice, we employed a linear projection of these CVs as these are convenient proxies for the nonlinear cosine function. Finally, the coordination number for the antibiotic–water interactions CN_CIP–WAT_ was used as a CV as well. All CV definitions and the associated parameters are provided in Table S3.

### Setup for Temperature Accelerated Sliced Sampling Simulations

To take advantage of the trimeric arrangement of the channels under investigation, we simultaneously applied three separate bias forces to study the antibiotic permeation through the three OmpF monomers, as employed in previous studies.^38,48^ The biased simulations were performed using the TASS method.^37^ Within this scheme, the principal CV *z* is sampled using a series of harmonic bias potentials in the range *z* ∈ [−2.4, 2.0] nm for the OmpE35 channel and *z* ∈ [−3.0, 2.0] nm for the OmpK35 channel. The simulation windows were generated in a stepwise fashion, wherein the final equilibrated configuration at position *i* – 1 was used as input configuration for the equilibration at umbrella position *i*. A 10 ns equilibration was performed at each umbrella position. Within each simulation window, we subsequently performed simulations using the harmonic potential bias (the same as was used for the equilibration runs) along the principal CV *z* and using temperature acceleration along the orthogonal CVs.^55,56^ Technically, in the TASS scheme, all biases are applied to a set of fictitious variables that are tightly coupled to the real CVs. These additional variables are introduced within an extended space that is maintained at a higher temperature. This scheme allows for the simultaneous inclusion of a large number of CVs, most recently demonstrated in a ligand dissociation study where up to 22 CVs were considered.^57^ For our simulations, the extended temperature was set to 900 K using a Langevin thermostat. Moreover, we prepared a total of 75 windows along the principal CV *z*. The windows were positioned 1.0 Å apart in the extracellular (EC) and periplasmic (PP) vestibules at both ends of the channel and 0.5 Å apart in the CR region. The values of the harmonic force constants range from 2000 kcal/mol at the channel ends to 6000 kcal mol^–1^nm^2^ in the CR in the case of the OmpK35 channel, and between 2000 and 5500 kcal/mol/nm^2^ for the OmpE35 channel. The small pore size in the CR restricts the free rotation of bulky solutes. Thus, such antibiotic molecules can only assume two possible orientations as they pass through the CR. In the case of CIP we either see the amino group ahead (orientation I) or the carboxyl group ahead (orientation II) while crossing the channel from the EC to the PP side (see Figure S3). During the generation of the initial configurations for the TASS sampling, care was taken to ensure that the antibiotic molecules are in orientation I in all umbrella windows that sample the CR. Orientations of the antibiotic molecule belonging to path II had to be sampled using an additional set of umbrella windows, wherein we ensured that the molecule is in orientation II during the equilibration step. Twenty-eight additional umbrella windows were employed to sample path II, while the obtained data was merged with that belonging to path I prior to the estimation of the free energy. The TASS simulations for the OmpE35 and OmpK35 porins altogether cumulated to simulation times of 27 and 30 μs, respectively.

### Estimation of Free Energy

The free energy surface (FES) estimation was performed using the TASS mean force approach as described previously.^38,58^ Individual 1D free energy estimates for each of the monomers were compared by suitably aligning the profiles to assess the convergence of the FES estimates from independent simulations. Note that the choice of the overlapping points for the free energy curves can affect the calculated error. In the present work, the alignment points roughly were chosen to yield the best fit between the independent estimates. Subsequently, the average 1D free energy profile from the three TASS simulations was obtained using a bootstrapping procedure based on the whole histogram bootstrapping method implemented in *g*_*wham*^59^ and employing the chosen overlap point to align the bootstrap estimates. As described previously,^38^ for a given umbrella position, the approach involves randomly picking a single histogram (with replacement) from *H* histograms (here, *H* is the number of independent samples; *H* = 3 for the present calculations). This procedure is used to pick *U* histograms (here, *U* is the number of umbrella windows employed for the TASS calculations) and the set is used to generate a single bootstrap estimate of the potential of mean force (PMF). For each histogram within a bootstrap sample, uncorrelated samples are obtained and the PMFs are estimated using the mean force TASS method. In this way, we can generate *X* bootstrap estimates that are used to obtain an average PMF and the associated error. For the present calculations, we used *X* = 100 bootstrap samples. The minimum free energy paths along the average 2D FES were determined using the zero-temperature string method.^48,53,60^

## Results and Discussion

### Structural Variations among OmpF Orthologs

As a start, we analyzed the structural variations among the different OmpF orthologs. While several crystal structures of OmpF have been available for quite a while already, structures for the OmpE35 and OmpK35 porins have only been reported more recently.^23^ The previous structural characterization based on all-atom MD simulations showed that OmpE35 has the same average pore radius in the CR of 3.1 Å at the CR of the OmpF channel.^23^ In contrast, OmpK35 has a wider radius at the CR of 3.6 Å. The wider pore radius of OmpK35 results in a slightly higher ion conductivity in electrophysiology experiments compared to OmpF and OmpE35.^23^ In the case of bulkier solutes such as antibiotic molecules, the permeation rates are governed by a large number of factors related to the channel and the respective molecule. Certainly, the structural and sequence variations among the orthologs are also expected to influence the relative permeation rates of a given antibiotic molecule. Moreover, keeping in mind the role of the hydrogen-bond network in the stabilization of the L3 loop and its connection to the antibiotic-induced loop dynamics, we were interested in how the sequence variations might influence the fluctuations of the L3 loop. The sequence alignment of OmpF with OmpE35 and OmpK35 shows that OmpE35 has a greater sequence identity of 77% (84% similarity) with OmpF than OmpK35 with 55% (67% similarity). If one considers only the residues in the CR, we find a sequence identity of 81% (90% similarity) between OmpE35 and OmpF. In the case of OmpK35, the sequence identity is 55% (72% similarity) for the same residues in the CR (Figure S3). Thus, in OmpE35 the CR has a large degree of residue conservation and most of the differences are present in the channel vestibules. For OmpK35, the divergence from OmpF is more pronounced and observed throughout the channel. Although there is a high degree of conservation between OmpE35 and OmpF in the CR, the divergence is greater between OmpK35 and OmpF. Note that the internal electric field is expected to vary based on the differences in the distribution of charged residues at the CR. The electrostatic potential across the constriction zone has been estimated to be 160 mV for OmpF and OmpE35 and 110 mV for OmpK35.^23^ This difference between OmpF (or OmpE35) and OmpK35 is possible considering the lower sequence similarity between the pores as stated earlier. The notable variations between the orthologs will be discussed below in more detail.

**Figure 2:**
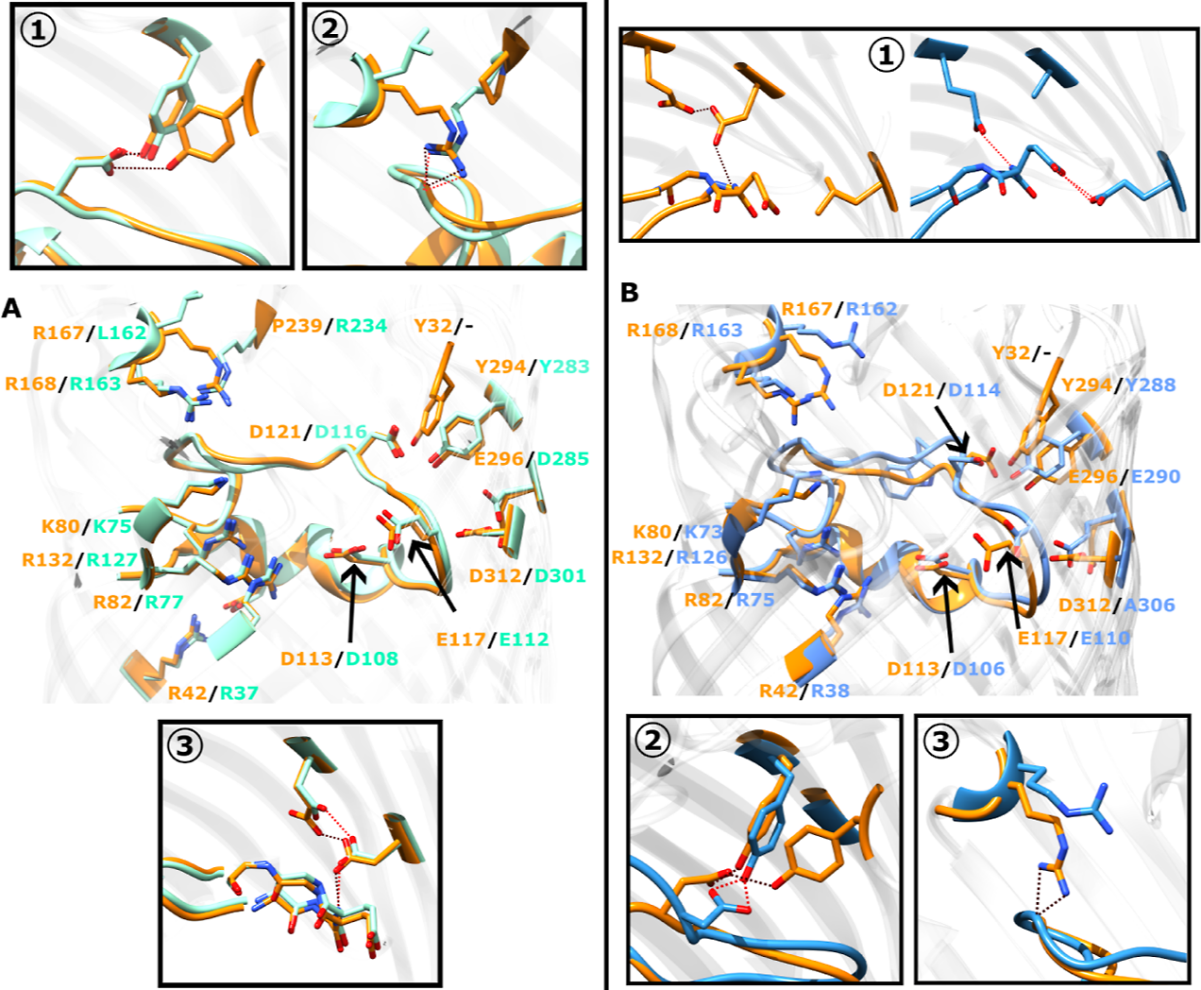
Structural superposition of the CRs of (A) the OmpE35 channel and (B) the OmpK35 channel with respect to the OmpF channel. The OmpF structure is depicted in orange, OmpE35 in green and OmpK35 in blue. Prominent residues in the constriction zone are highlighted. The residue labels are colored in the same color code as the respective structures. The insets zoom into differences in the residues that provide stabilizing effects to the L3 loop by hydrogen bonds.

Figure 2A shows the structural superposition of OmpF and OmpE35 crystal structures, focusing on the prominent residues in the CR. The conformation of loop L3 is very similar in both orthologs. However, we find differences in the residues that form stabilizing interactions with the loop. The most prominent difference is the loss of the hydrogen bond D121-Y32 that is present in OmpF. Such an interaction with the equivalent D116 residue is missing in OmpE35 due to a significant shortening of the loop L1 and a loss of the tyrosine residue (see Figure 2A, inset 1). The loss of this stabilizing interaction can result in larger fluctuations in the flexible L3-FS segment (residues F113-S120) of OmpE35. Note that the residue D121 in OmpF also forms another hydrogen bond with Y294 residue. This hydrogen bond is retained in OmpE35 in the form of the D116-Y283 interaction. This apart, we also observed a substitution of the arginine residue R167 in OmpF to the hydrophobic leucine L162 in OmpE35. The R167 residue forms a hydrogen bond with the L3 backbone at residue S125 in OmpF. Figure 2A, inset 2, shows this part of the protein in OmpE35, a compensatory mutation in the form of residue R234, however, replaces the function of R167. Finally, the backbone of the L3 tip is stabilized in OmpF by a network of hydrogen bonds involving residues E296 and D312. In OmpE35, a similar stabilizing network is maintained by the residues D286 and D301 (see Figure 2A, inset 3). Overall, the loop conformations and dynamics in OmpE35 are expected to be similar to those in OmpF with a potentially slightly greater flexibility of the L3-FS segment in the former.

The structural superposition of OmpK35 crystal structure with that of OmpF shows larger variations in critical residues in the CR (see Figure 2B). Comparing the structure of the L3 loop, one immediately notices differences in the form of a shift at the L3 tip at position 110 and a larger loop bulge due to the insertion of a tryptophan residue at position 116 of OmpK35. Moreover, the side chain of residue W116 interacts with the adjacent barrel wall, possibly leading to a stabilizing effect on the L3-FS segment in OmpK35. In OmpK35, the observed shift of the L3 tip toward the barrel wall appears to be due to a difference in the residues that stabilize the tip. Figure 2B, inset 1, shows that in the case of OmpK35, the L3 backbone is stabilized directly by the residue E290 rather than through a network formed by E296 and D312 as observed in OmpF. The loss of the aspartate residue in OmpK35 leads to a shift of the L3 tip toward the residue E290. Additional stabilization to the L3 tip in this position is achieved through the interactions between the residues E110 and E20. Due to the proximity of the residues E110 and E20, one of the residue has a high probability to be in a protonated state that enables a hydrogen bond interaction (see Section S1). Altogether, these differences result in an increase in the pore radius of the OmpK35 channel. Other key differences are in the hydrogen bonds that stabilize the L3-FS segment. Similar to OmpE35, in OmpK35 we note a loss of a tyrosine residue located on loop L1 stabilizing L3-FS (see Figure 2B, inset 2). At the same time, the D114-Y288 interaction is retained, which is equivalent to the D121-Y294 interaction in OmpF. Another difference between OmpF and OmpK35 is that the residue R162 (equivalent to R167 in OmpF) does not form a hydrogen bond with the backbone of loop L3 (see Figure 2B, inset 3). While these differences are expected to increase the flexibility of the L3-FS segment in OmpK35, the W123 interaction with the barrel wall might compensate and provide additional stability to the L3-FS segment.

**Figure 3:**
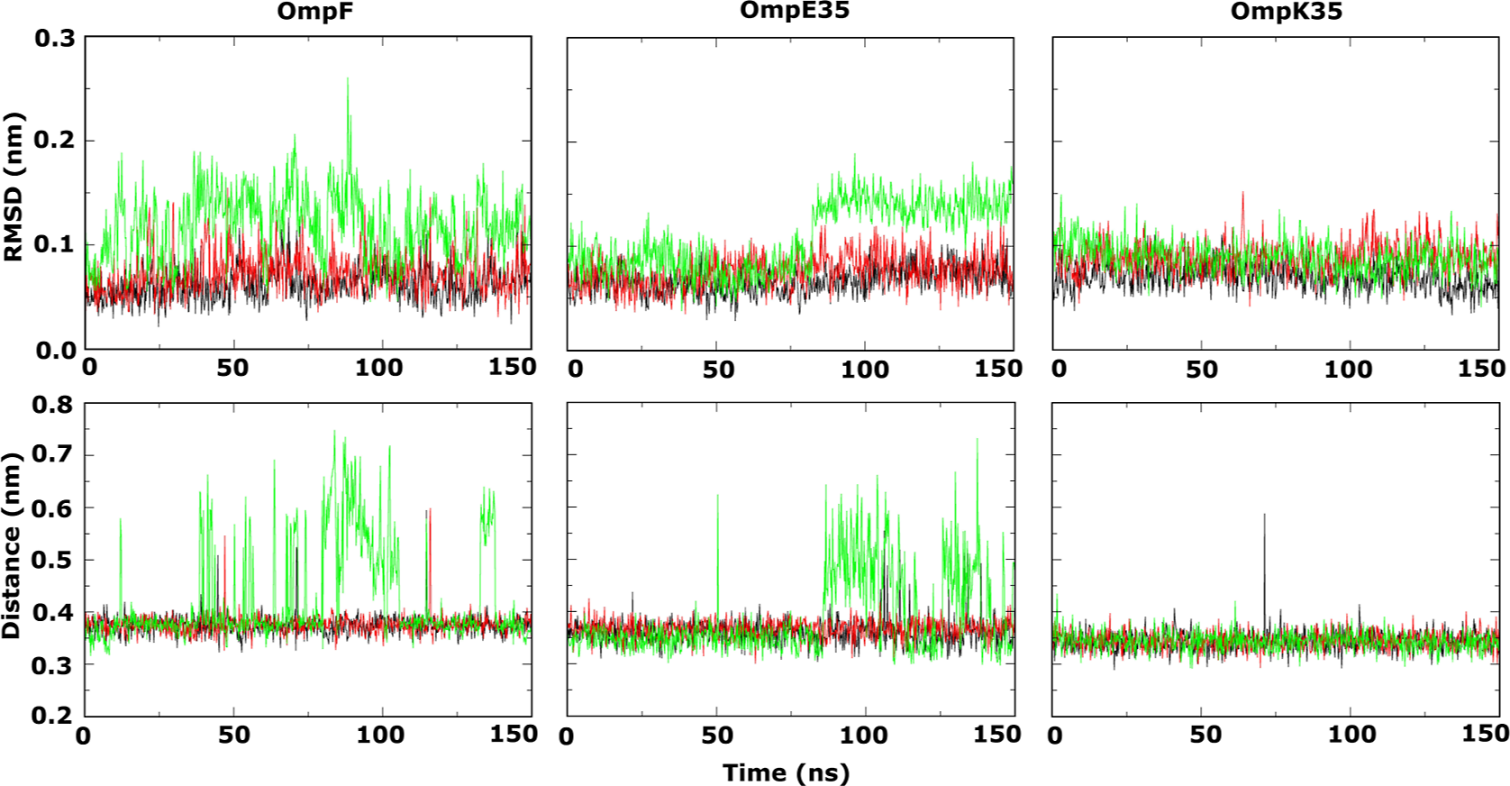
(Top row) Root mean square deviation of the C_α_ atoms of the residues within the L3-FS region of OmpF, OmpE35, and OmpK35 calculated based on a 150 ns-long unbiased all-atom MD simulation. L3-FS corresponds to the L3 loop segment F118 to S125 in OmpF, F113 to S120 in OmpE35 and W111 to T119 in OmpK35. (Bottom row) Plots for the hydrogen bond distances for the D121-Y294 bond (in OmpF) and its equivalent hydrogen bonds, i.e, D116-Y283 in OmpE35 and D114-Y288 in OmpK35. All analyses were performed on the three monomers individually, as depicted by the different colors.

To examine if the aforesaid variations affect the L3-FS stability, we performed unbiased simulations of these channels. Figure 3 depicts plots of the root-mean-square deviation (RMSD) for the L3-FS region, showing that in OmpE35 this segment shows a large propensity for backbone fluctuations similar to that of OmpF. In contrast, the L3-FS segment in OmpK35 is found to be quite stable in the unbiased simulations. In addition to the backbone fluctuations, the L3-FS stabilizing hydrogen bond in OmpE35 (D116-Y283) also undergoes fluctuations. The corresponding hydrogen bond in OmpK35 (D114-Y288) however remains stable throughout. The pore radii calculated from these trajectories at the narrowest section of the pore is about 3.32 Å in case of OmpF and OmpE35, and about 3.59 Å in case of OmpK35 (Figure S5). The pore dynamics leads to fluctuations around these mean values by about 0.21 Å in all cases. However, calculations of the pore radii in the region around the L3-FS segment lying in the preorientation region (PR) shows larger fluctuations of 0.34 Å in OmpF, 0.30 Å in OmpE35, and of only 0.18 Å in OmpK35. Overall, these results indicate that the variations in and around the CR significantly influence the fluctuations of the pore size and the stability of loop L3.

### Free Energy Calculations for CIP Permeation Suggests Faster Permeation through OmpK35

**Figure 4:**
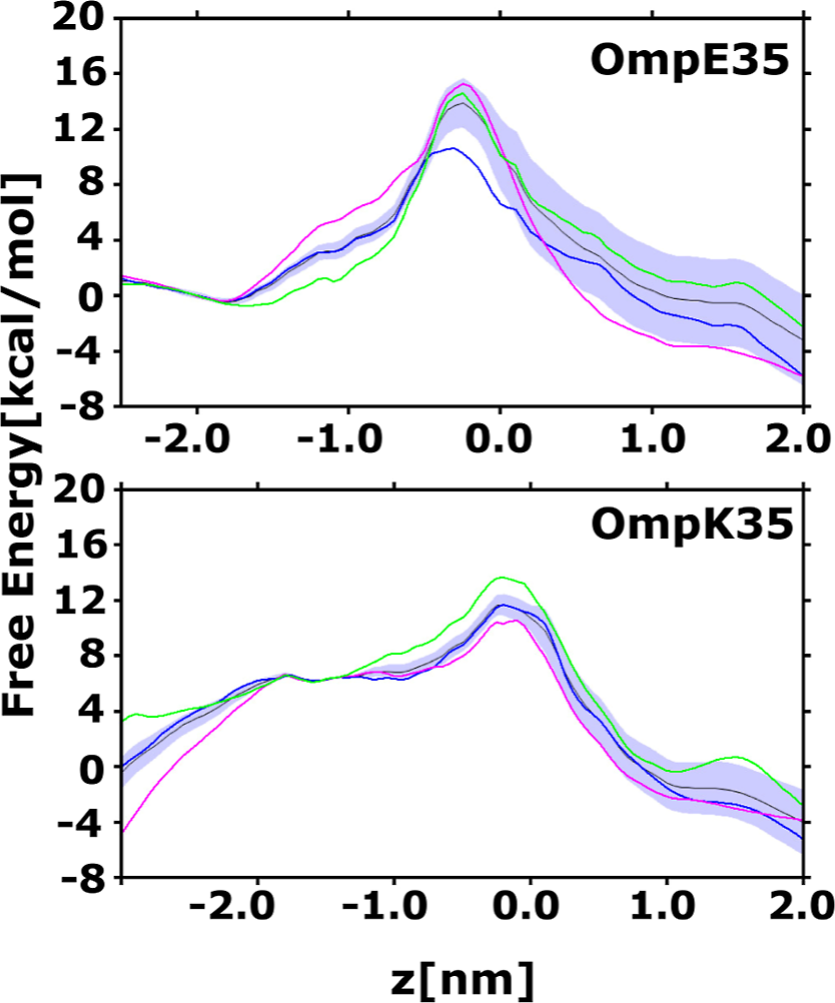
One-dimensional free energy plots for CIP permeation through OmpE35 and OmpK35 calculated using TASS simulations. The principal CV, *z* is the projection of the center of mass distance between CIP and channel monomer, along the *z*-axis. The free energy estimates for permeation through the three individual monomers are shown in blue, green and magenta. The average free energy (in black) estimate and associated standard error (shaded region) were calculated using a histogram bootstrapping approach.

**Figure 5:**
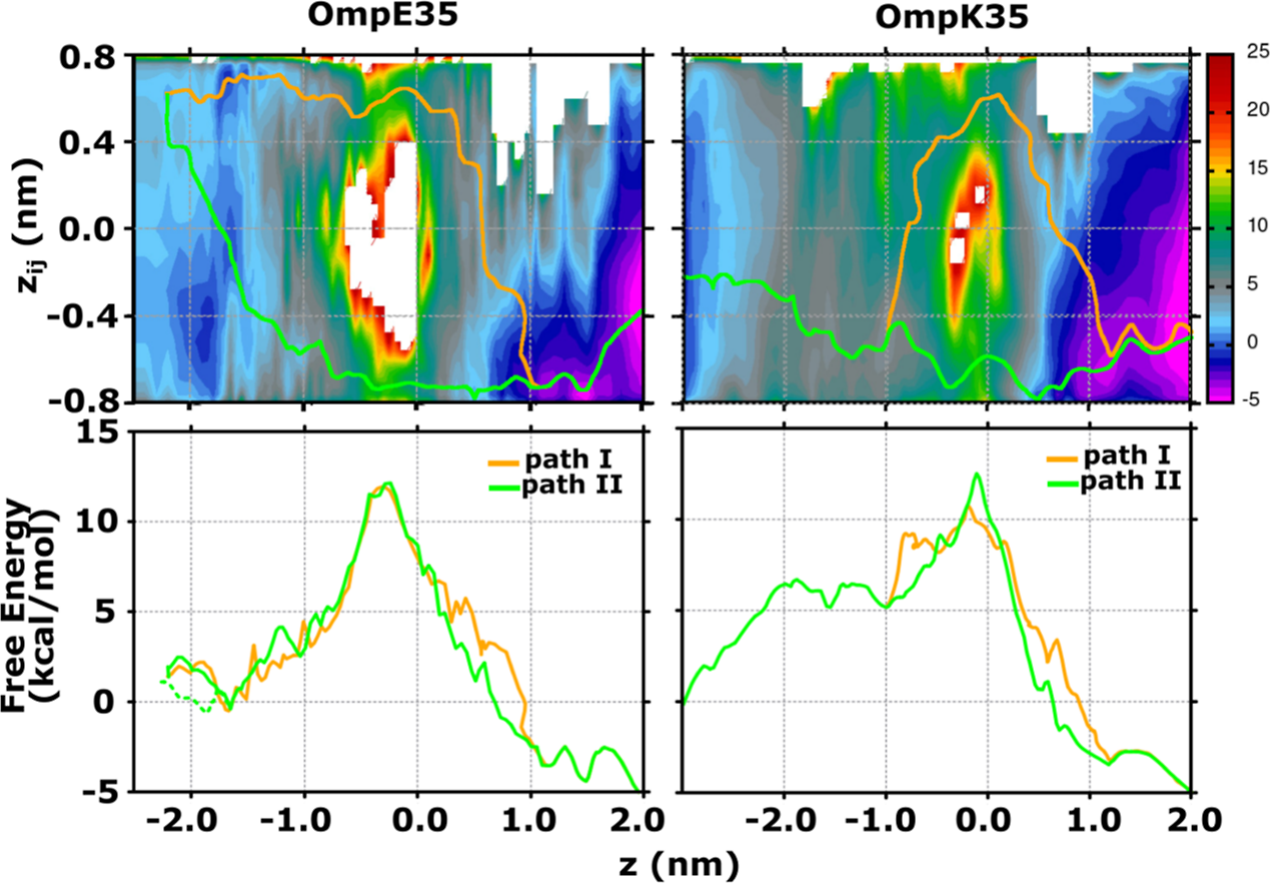
Two-dimensional free energy estimates for CIP permeation through OmpE35 and OmpK35 are shown in the upper panels. The CV *z* is the projection of the center of mass distance between CIP and channel monomer along the *z*-axis, and the CV *z*_*ij*_ is the projection of the longest axis of CIP along the *z*-axis. The two possible permeation paths, I and II, are calculated using a zero-temperature string method. The free energy along the two paths is depicted in the lower panels. The respective plots for OmpF are shown in Figure S9.

The 1D and 2D FES for CIP permeation through OmpE35 and OmpK35 are depicted in Figures 4 and 5 similar to those in Figure S9 for OmpF. The 1D FES calculated along the CV *z* suggests that the permeation barrier for CIP in the case of OmpK35 (11.8 ± 1.16 kcal/mol) and OmpE35 (12.9 ± 1.77 kcal/mol) are in a similar range. However, due to the larger pore diameter in OmpK35, it is expected that there would be a relaxation in the steric restrictions to permeation and possibly a lower barrier to permeation. The 2D FES provides a more detailed view of the permeation with additional information on the orientation, *i.e.*, through CV *z*_*ij*_. The 2D-FES plots in Figure 5 shows that the CIP molecule can permeate *via* two possible pathways in both OmpE35 and OmpK35. The two pathways are related to the two possible orientations a bulky antibiotic can attain in the CR: one with the amino group going ahead (path I) and the other with the carboxylate group going ahead (path II) as the antibiotic traverses the CR. For OmpE35, however, the 2D-FES has a significant undersampled region within the configuration space. This region is an entropically forbidden region of the space that appears due to the narrow diameter at the center of the channel. Such a feature was also present in the 2D-FES estimates in previous studies on the OmpF channel.^38,61^ Notably, in the case of OmpK35 this forbidden region is significantly reduced. This result was to be expected, considering the larger minimum pore diameter in the CR of the OmpK35 channel compared to that of OmpE35 (see Figure S4). Furthermore, we calculated the minimum free energy path associated with paths I and II using a zero-temperature string method, as also shown in Figure 5. A comparison of the free energy for the translocation through OmpE35 along path I and path II suggests that for both paths, the molecule encounters a free energy barrier of around 12 kcal/mol. Thus, permeation can occur *via* either of the two paths with similar probabilities. For OmpK35, we find that path I has a greater feasibility due to a lower barrier of 10.5 kcal/mol compared to path II with a barrier height of 13 kcal/mol. It must be pointed out that in the case of OmpF, path I was found to be energetically more feasible due to a lower barrier of 11.5 kcal/mol compared to that of path II with 13.5 kcal/mol.^31^ Considering the high sequence identity between OmpF and OmpE35, especially for the residues in the CR, the difference in barriers seems unexpected. With the reported error in free energy of >1.0 kcal/mol it is not possible to conclusively comment of the relative difference in the CIP permeation rates between OmpF and OmpE35 based on the free energy values.

**Figure 6:**
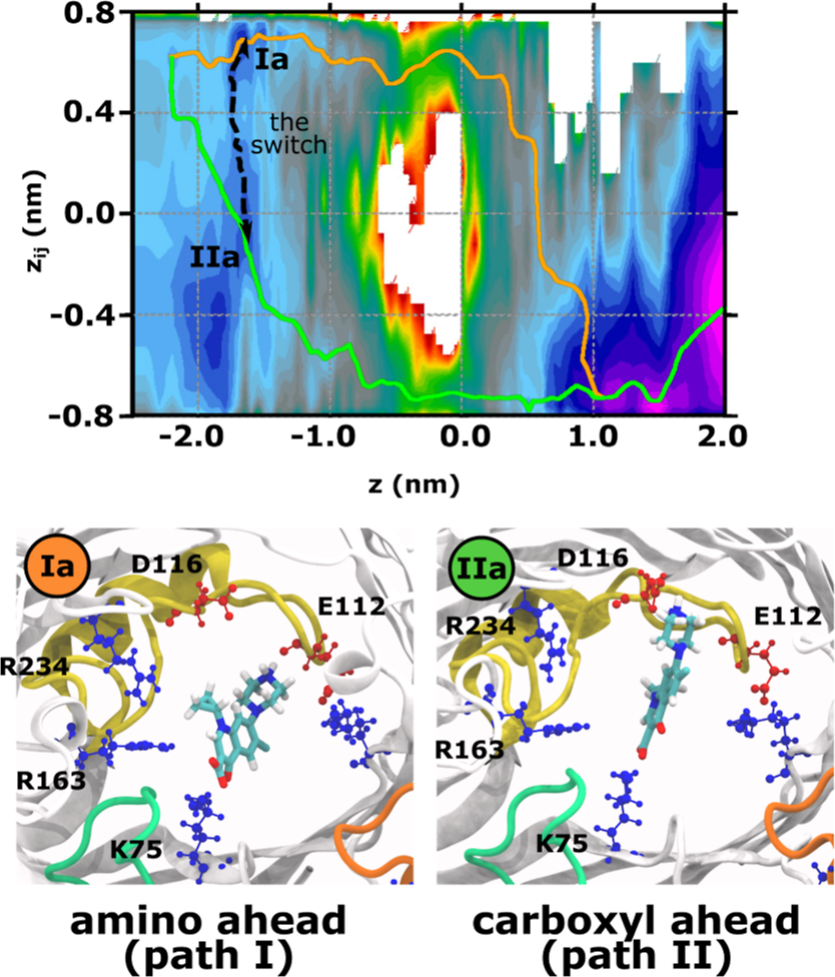
Path switching point in the PR of OmpE35 that allows the CIP molecule to transition between path I and path II configurations during permeation. The black arrow in the upper panel shows the switching path along the 2D-FES estimated using TASS. The lower panels depict the prominent conformations involved in the switch between path I and path II. The L3 loop is shown in yellow and the charged residues are labeled.

Next, we examined the sampled CIP configurations within the two channels to obtain a molecular picture of the permeation process. Antibiotic molecules can assume a myriad of configurations in the wide EC vestibule. However, the channel gets narrower toward the CR, thus limiting the accessible configurational space. Moreover, within the EC region toward the CR, also termed the PR, a molecule with an internal dipole preferentially aligns with the electric field transverse to the pore axis. This PR region lies roughly in the range z ∈ [−1.6, −0.5] nm. Notably, simulations of CIP permeation through OmpF showed that the PR serves as a region for a possible path-switching maneuver, where the molecule can switch from path I to path II and vice versa.^31^ As shown in Figure 6 for OmpE35, we find a similar switching region involving a transition from position **Ia** where the CIP molecule interacts with K75 and D116 to position **IIa** in which the molecule interacts with residues K75 and E112. This transition involves a shift of the piperazine amine group of CIP from D116 to E112 with the K75 interactions with the carboxylate moiety acting as a pivot. From here on, the molecule passes through the CR either *via* path I or path II. Figure S6 shows prominent poses of CIP as it crosses the CR *via* path I and path II from **Ia** to **If** and **IIa** to **IIf**, respectively. The configurations along the two paths are similar to those previously observed for CIP permeation through OmpF.^31^ The CIP molecule moves through the CR along a track of positively charged residues on one side and negatively charged residues on the other, either in the orientation belonging to path I or to path II, and subsequently exits the CR.

For the OmpK35 channel, the PR was largely populated by states with orientations corresponding to path II, as is also apparent from the 2D-FES in Figure 5. As the molecule enters the CR, it reorients to align with the internal electric field (Figure S7, pose **P1**). However, we find that in the case of OmpK35 the CIP molecule enters further into the CR along path II before possibly undergoing a transition toward path I. The switching transition is depicted in Figure S7 as poses **P2** to **P5**. The charged amine group of CIP that initially interacts with D114 residue in pose **P2**, undergoes a transition to interact with the E110 residue as shown in poses **P3** and **P4** and finally shifts to pose **P5** where it interacts with residue D106, completing the switch to path I. This late switch in the CR is feasible due to the wider pore in OmpK35. Thereafter, the molecule crosses and exits the CR along path I as shown in poses **P6** to **P9**.

### Antibiotic Induced L3-FS Conformational Dynamics in OmpF Orthologs

**Figure 7:**
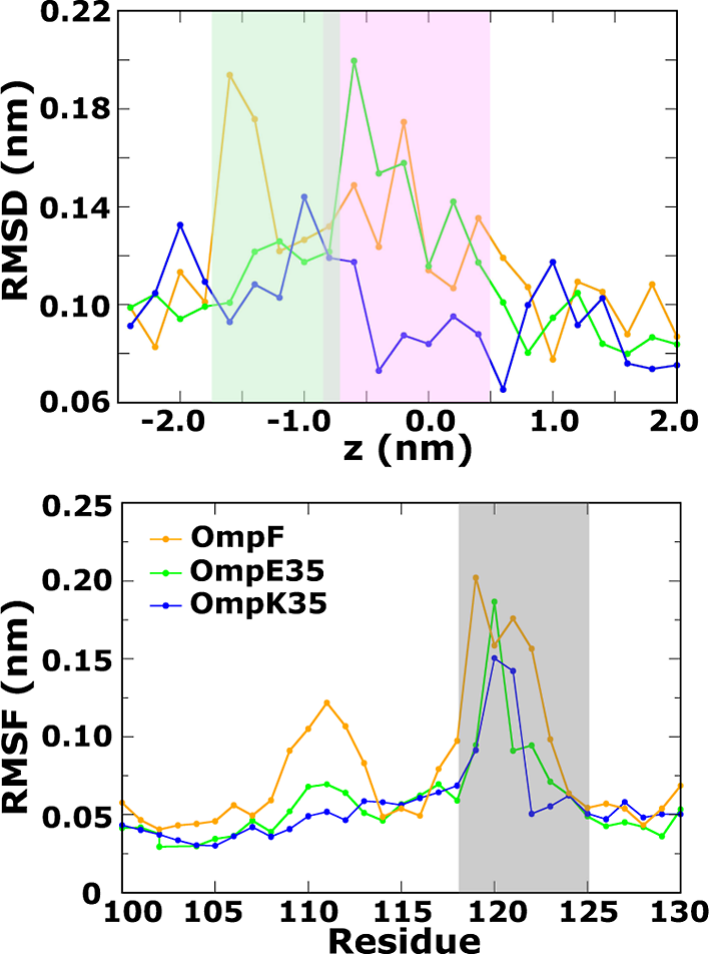
Fluctuations of the L3-FS segment during CIP translocation through OmpF (yellow), OmpE35 (green) and OmpK35 (blue). L3-FS corresponds to the L3 loop segment F118 to S125 in OmpF, F113 to S120 in OmpE35 and W111 to T119 in OmpK35. The upper panel shows the C_α_ RMSD values calculated for the L3-FS region from the TASS trajectories sampling CIP configurations at different positions along the channel. The lower panel depicts the per residue RMSF values for the backbone of loop L3 calculated from the simulation windows sampling the CR. The residue number follows the numbering in the OmpF porin. Note that the L3 loop of OmpK35 has an additional insertion in the L3-FS region at position 116. This residue has been omitted in the RMSF plot.

A key feature of the CIP permeation mechanism through the porin OmpF was the observed L3 dynamics, particularly in the L3-FS segment, associated with permeation.^31^ From unbiased simulations, even in the absence of an antibiotic molecule, the L3-FS of OmpF already shows some backbone fluctuations as well as fluctuations in the hydrogen bonds that stabilize the L3-FS segment as shown in Figure 3. Given this observation, antibiotic-induced changes in the L3-FS conformation are to be expected. The L3-FS segment of OmpE35 behaves similarly to that in OmpF in unbiased simulations. Thus, CIP-induced L3-FS dynamics appears to be feasible. Our analysis of the TASS trajectories shows that the passage of CIP through the PR and CR is associated with L3-FS fluctuations, as can be discerned from the L3-FS RMSD plot in Figure 7. While the unbiased simulations for OmpK35 show a stable loop (see Figure 3), the TASS trajectories show that in this case as well, the L3-FS undergoes induced conformational fluctuations. However, in comparison to OmpF and OmpE35, we see smaller conformational fluctuations associated with the permeation event based on the lower RMSD values. A structural analysis suggests that the subdued L3-FS backbone fluctuation in OmpK35 can be attributed to the stabilizing effect of the indole ring of the W116 residue interacting with the barrel wall. This stabilizing effect can be seen in the RMSF plot in Figure 7, where the RMSF values decrease sharply in the case of OmpK35 for residues from position 122 onward. Note that residue W116 is actually an insertion that has been omitted in the RMSF plot, but corresponds to the position between residues 122 and 123 according to the OmpF numbering used in the plot. This apart, the stability of the L3-FS segment in OmpK35 is also apparent from the results of the unbiased simulations in Figure 3. The RMSF plot of OmpE35 is interesting as well, as it shows that the loop fluctuations are limited to the L3-FS (shaded in gray). In contrast for OmpF, one can also see a peak in the region around the residues 112 to 114. In the OmpF study,^31^ this peak was attributed to conformational changes in the residue D113 that also provides key interactions to the amine moiety of the CIP molecule during translocation. It is interesting to note that fluctuation in this residue is marginal in the equivalent D108 residue of OmpE35 and more so in the case of the D106 residue of OmpK35. In the case of OmpK35, such a difference may be due to the wider pore that enables passage of CIP without the need for a conformational transition of the D106 residue. However, OmpE35 is closely related to OmpF and has a similar pore diameter. In the analysis of trajectories, we only found marginal fluctuations in the D108 side chain associated with the passage of the CIP molecule. Overall, we note that the conformational dynamics induced by the antibiotic molecule is also an important factor in the permeation mechanism through OmpF orthologs. Moreover, the extent of the loop dynamics associated with a translocation event depends on the particular channel and in particular on the stabilization of loop L3.

## Conclusion

Experimental studies have examined the structural and physicochemical aspects of the process of antibiotic influx into Gram-negative bacterial cells. Early studies on the permeation of a range of antibiotics revealed a notably higher permeation rate for zwitterionic antibiotics than for mono- and dianionic antibiotics.^7,16^ The investigations suggested a dominant influence of solute charge distribution, hydrophobicity, and size in determining the effective permeation rate through porins. Zwitterionic antibiotics have also been found to have a stronger binding in the CR than anionic antibiotics.^18^ Electrostatics plays a major role in the permeation as has been suggested by the observations of strong current blockages in electrophysiology studies that indicate the presence of binding sites in the CR,^62^ through the observed binding site in the OmpF structure cocrystallized with ampicillin,^11^ and in biased metadynamics simulations of various antibiotics wherein the most prominent affinity sites involve interactions with charged residues in the CR.^18,27,29,48,53,63^ Prominently, a systematic study of different OmpF orthologs and a representative set of β-lactam antibiotics suggested that a successful permeation involves achieving a balance in the electrostatic and steric factors.^23^ Notably, this study suggested a scoring function that takes into account the statistical averages of various channel and antibiotic properties, as well as their thermal fluctuations. Later on, detailed biased simulations of OmpF with the zwitterionic ciprofloxacin molecule highlighted the possibility of antibiotic-induced fluctuations of the L3 loop at the pore CR.^31,38^ Based on these results, it was suggested that the permeation is not only dependent on thermal fluctuations about the statistical averages but perhaps more critically on the induced fluctuations of the L3 loop during antibiotic passage through the CR. However, a direct confirmation of the role of loop backbone fluctuations through mutations in the L3 loop is not straightforward. Previous studies have shown that the channel permeation properties are sensitive to mutations and any mutation to restrict backbone fluctuations in the L3-FS would affect other properties such as pore size and the electrostatics.^24,64^ A study on molecules with different charge distributions, it was reported that that the antibiotic-induced fluctuations are observed only in zwitterionic and cationic antibiotics with a positive charge that is accessible for interactions with the negatively charged residues of the L3 loop.^36^ It is worth mentioning that in the case of enrofloxacin (ENR), which differs from CIP in that the former has an ethyl cap on the positively charged amine group, interactions of the amine group in ENR with the negatively charged residues of the L3-FS are not feasible due to steric restriction, and thus the molecule does not induce loop fluctuations. Besides this, the role of the conformational flexibility of an antibiotic and the accessible conformational space at the CR is bound to another dominating factor, and is possibly intimately related to the empirically deduced role of the number of internal rotatable bonds in the antibiotic molecule.^32^

The present study aimed at extending our current molecular-level understanding of the permeation process and the role of antibiotic-induced pore fluctuations during permeation through OmpF orthologs. More specifically, the objective was to see if the induced L3 dynamics is a general feature of all porins or if it is determined by ortholog-specific structural variations. To this end, we first discussed the differences in sequence and structure between OmpF, OmpE35, and OmpK35 and how these variations might affect the permeation process. The observed variations suggest possible differences in the pore dynamics and the extent of L3 stabilization, a finding that was also supported by unbiased simulations of the three porins. To further examine how these differences influence the permeation of a given antibiotic, we studied the permeation of the antibiotic CIP through the porins OmpE35 and OmpK35, while OmpF was already investigated in an earlier study.^31^ The simulations indicate that the observed differences in the pore structure, *i.e.*, in the minimum pore radius and in the L3 stabilization, influence the feasibility of the two possible CIP orientations during translocation. The difference is particularly striking between OmpF (or OmpE35) and OmpK35 in terms of the observed structural variations, L3 dynamics, and the permeation mechanism. In OmpK35, an additional stabilization of the unstructured L3-FS segment leads to greater stability and rigidity both in the absence and presence of the zwitterionic antibiotic molecule. In OmpF, the transient conformation fluctuations of L3-FS induced by an antibiotic molecule containing a positive charge were suggested to aid the permeation of bulky antibiotics by reducing the entropic contribution to the barrier. In OmpK35, however, the greater rigidity of the loop appears to diminish the mechanistic role of L3-FS dynamics in permeation processes. At the same time, the larger pore radius of OmpK35 makes up for the loss of the L3-FS flexibility. Energetically, CIP has a translocation barrier through OmpK35 of 10.5 kcal/mol which is smaller than that for OmpF with 11.5 kcal/mol and for OmpE35 with 12 kcal/mol. This result is in line with the trend previously reported for penicillins^65^ while no experimental trend has been reported so far for the antibiotic CIP. We speculate however that the differences in the permeation characteristics through OmpF (or OmpE35) and through OmpK35 may be different in case of bulkier zwitterionic antibiotics. A larger pore size in OmpK35 from *K. pneumoniae* compared to OmpF from *E. coli* may not always result in a faster permeation of antibiotics through the former. As the size of the antibiotic molecule increases, the OmpK35 pore would present a higher permeation barrier, while the greater rigidity of the L3-FS segments suggests that antibiotic-induced L3 dynamics would not play a dominant role in the translocation process. For such bulkier zwitterionic drugs, the OmpF pore could still present a more efficient permeation path than the OmpK35 pore. For such bulkier drugs, it may be advantageous to contain internal rotatable bonds for more flexibility. In the present work, we have not studied bulkier drugs through MD simulations due to significant sampling issues with increasing size of the solute under investigation.^66^ Obtaining converged estimates for free energy still remains a challenge even in case of antibiotics of modest sizes (200–400 Da) and methodological developments to tackle more complicated systems is an active area of work.^38,67−69^ Based on the studies thus far, the TASS method does present itself as a suitable method to study complex systems. Work in the direction of extending the investigations to larger antibiotic molecules is in progress. Nonetheless, the insights obtained from the present simulations can help future computational investigations on antibiotic permeation through these channels. The present as well as recent investigations on porins using the TASS scheme have focused only on the role of the channel in antibiotic permeation and did not study the effect of lipopolysaccharides (LPS) on the extracellular leaflet of the outer membrane. Specific patterns of LPS interactions with porins have been observed in long simulations^70^ and affect the channel orientation, extracellular loop dynamics and transport energetics of solutes.^71−74^ It would be interesting to compare the permeation energetics and channel dynamics during antibiotic permeation in the presence of modeled LPS on the EC side. A recent study reports that LPS does not markedly influence the internal electric field at the porin constriction, a dominant factor influencing permeation.^75^ However, the reported differences in the dynamics of loop L3 and of the extracellular loops of the channel in the presence and absence of LPS imply a significant influence on the effective permeation rates.

In the context of understanding the permeation in the case of the bacterium *K. pneumoniae*, further studies would need to also focus on the OmpK36 channel, which has an important physiological role in the survival of the pathogenic strains. Interestingly, mutations in the L3 loop of OmpK36 have been reported that improve the fitness of the pathogenic strains of *K. pneumoniae*.^76^ While data on accumulation and MIC values of different antibiotics is available, the role of efflux rates complicates the derivation of correlations between antibiotic properties and accumulation. Furthermore, most of the studies on permeation have focused on OmpF as a model system. Previous investigations, for instance, have looked to decouple the influx and efflux process and study the accumulation of antibiotics and identified antibiotic substituents that are critical determinants for permeation through OmpF.^13^ Similar studies that systematically look at permeation rates of antibiotics with different sizes and charge profiles through the orthologs systems may be necessary to further understand the differences in the permeation behavior of antibiotics among the various orthologs.

## Supporting Information Available

Additional analysis of protonation states and of the MD simulations are provided (PDF)

## References

[ref1] Heesterbeek D. A.; Muts R. M.; van Hensbergen V. P.; de Saint Aulaire P.; Wennekes T.; Bardoel B. W.; van Sorge N. M.; Rooijakkers S. H. Outer Membrane Permeabilization by the Membrane Attack Complex Sensitizes Gram-Negative Bacteria to Antimicrobial Proteins in Serum and Phagocytes. PLoS Pathog. 2021, 17, e1009227 10.1371/journal.ppat.1009227. 33481964 PMC7886145

[ref2] World Health Organization. Global Antimicrobial Resistance and Use Surveillance System (glass) Report 2022; WHO, 2022.

[ref3] Mortimer P. G. S.; Piddok L. J. V. The Accumulation of Five Antibacterial Agents in Porin-Deficient Mutants of *Escherichia coli*. J. Antimicrob. Chemother. 1993, 32, 195–213. 10.1093/jac/32.2.195. 8226422

[ref4] Pavlova A.; Hwang H.; Lundquist K.; Balusek C.; Gumbart J. C. Living on the Edge: Simulations of Bacterial Outer-Membrane Proteins. Biochim. Biophys. Acta, Biomembr. 2016, 1858, 1753–1759. 10.1016/j.bbamem.2016.01.020. 26826270

[ref5] Im W.; Khalid S. Molecular Simulations of Gram-Negative Bacterial Membranes Come of Age. Annu. Rev. Phys. Chem. 2020, 71, 171–188. 10.1146/annurev-physchem-103019-033434. 32070216

[ref6] Prajapati J. D.; Kleinekathöfer U.; Winterhalter M. How to Enter a Bacterium: Bacterial Porins and the Permeation of Antibiotics. Chem. Rev. 2021, 121, 5158–5192. 10.1021/acs.chemrev.0c01213. 33724823

[ref7] Yoshimura F.; Nikaido H. Diffusion of Beta-lactam Antibiotics through the Porin Channels of *Escherichia Coli* K-12. Antimicrob. Agents Chemother. 1985, 27, 84–92. 10.1128/AAC.27.1.84. 2580479 PMC176210

[ref8] Nikaido H. Molecular Basis of Bacterial Outer Membrane Permeability Revisited. Microbiol. Mol. Biol. Rev. 2003, 67, 593–656. 10.1128/MMBR.67.4.593-656.2003. 14665678 PMC309051

[ref9] Pages J. M.; James C. E.; Winterhalter M. The Porin and the Permeating Antibiotic: A Selective Diffusion Barrier in Gram-Negative Bacteria. Nat. Rev. Microbiol. 2008, 6, 893–903. 10.1038/nrmicro1994. 18997824

[ref10] Dhakshnamoorthy B.; Ziervogel B. K.; Blachowicz L.; Roux B. A Structural Study of Ion Permeation in OmpF Porin from Anomalous X-ray Diffraction and Molecular Dynamics Simulations. J. Am. Chem. Soc. 2013, 135, 16561–16568. 10.1021/ja407783a. 24106986 PMC3868647

[ref11] Ziervogel B. K.; Roux B. The Binding of Antibiotics in OmpF Porin. Structure 2013, 21, 76–87. 10.1016/j.str.2012.10.014. 23201272 PMC3545085

[ref12] Zgurskaya H. I.; Weeks J. W.; Ntreh A. T.; Nickels L. M.; Wolloscheck D. Mechanism of Coupling Drug Transport Reactions Located in Two Different Membranes. Front. Microbiol. 2015, 6, 100 10.3389/fmicb.2015.00100. 25759685 PMC4338810

[ref13] Vergalli J.; Atzori A.; Pajovic J.; Dumont E.; Malloci G.; Masi M.; Vargiu A. V.; Winterhalter M.; Réfrégiers M.; Ruggerone P.; et al. The Challenge of Intracellular Antibiotic Accumulation, a Function of Fluoroquinolone Influx Versus Bacterial Efflux. Commun. Biol. 2020, 3, 198 10.1038/s42003-020-0929-x. 32346058 PMC7189378

[ref14] Rybenkov V. V.; Zgurskaya H. I.; Ganguly C.; Leus I. V.; Zhang Z.; Moniruzzaman M. The Whole Is Bigger Than the Sum of Its Parts: Drug Transport in the Context of Two Membranes with Active Efflux. Chem. Rev. 2021, 121, 5597–5631. 10.1021/acs.chemrev.0c01137. 33596653 PMC8369882

[ref15] Parkin J.; Khalid S. Atomistic Molecular-Dynamics Simulations Enable Prediction of the Arginine Permeation Pathway through OccD1/OprD from Pseudomonas-aeruginosa. Biophys. J. 2014, 107, 1853–1861. 10.1016/j.bpj.2014.08.035. 25418166 PMC4213661

[ref16] Nikaido H.; Rosenberg E. Y.; Foulds J. Porin Channels in Escherichia Coli: Studies with Beta-Lactams in Intact Cells. J. Bacteriol. 1983, 153, 232–240. 10.1128/jb.153.1.232-240.1983. 6294048 PMC217361

[ref17] Kojima S.; Nikaido H. Permeation Rates of Penicillins Indicate That Escherichia Coli Porins Function Principally As Nonspecific Channels. Proc. Natl. Acad. Sci. U.S.A. 2013, 110, E2629–E2634. 10.1073/pnas.1310333110. 23798411 PMC3710850

[ref18] Danelon C.; Nestorovich E. M.; Winterhalter M.; Ceccarelli M.; Bezrukov S. M. Interaction of Zwitterionic Penicillins with the OmpF Channel Facilitates Their Translocation. Biophys. J. 2006, 90, 1617–1627. 10.1529/biophysj.105.075192. 16339889 PMC1367313

[ref19] Matsumura N.; Minami S.; Watanabe Y.; Iyobe S.; Mitsuhashi S. Role of Permeability in the Activities of β-Lactams against Gram-Negative Bacteria Which Produce a Group 3 β-Lactamase. Antimicrob. Agents Chemother. 1999, 43, 2084–2086. 10.1128/AAC.43.8.2084. 10428944 PMC89422

[ref20] Samanta S.; Bodrenko I.; Acosta-Gutiérrez S.; D’Agostino T.; Pathania M.; Ghai I.; Schleberger C.; Bumann D.; Wagner R.; Winterhalter M.; et al. Getting Drugs through Small Pores: Exploiting the Porins Pathway in *Pseudomonas aeruginosa*. ACS Infect. Dis. 2018, 4, 1519–1528. 10.1021/acsinfecdis.8b00149. 30039960

[ref21] Vergalli J.; Bodrenko I. V.; Masi M.; Moynié L.; Acosta-Gutiérrez S.; Naismith J. H.; Davin-Regli A.; Ceccarelli M.; Van Den Berg B.; Winterhalter M.; et al. Porins and Small-Molecule Translocation across the Outer Membrane of Gram-Negative Bacteria. Nat. Rev. Microbiol. 2020, 18, 164–176. 10.1038/s41579-019-0294-2. 31792365

[ref22] Lou H.; Chen M.; Black S. S.; Bushell S. R.; Ceccarelli M.; Mach T.; Beis K.; Low A. S.; Bamford V. A.; Booth I. R.; et al. Altered Antibiotic Transport in OmpC Mutants Isolated from a Series of Clinical Strains of Multi-drug Resistant *E. Coli*. PLoS One 2011, 6, e25825 10.1371/journal.pone.0025825. 22053181 PMC3203869

[ref23] Acosta-Gutiérrez S.; Ferrara L.; Pathania M.; Masi M.; Wang J.; Bodrenko I.; Zahn M.; Winterhalter M.; Stavenger R. A.; Pagès J. M.; et al. Getting Drugs into Gram-Negative Bacteria: Rational Rules for Permeation through General Porins. ACS Infect. Dis. 2018, 4, 1487–1498. 10.1021/acsinfecdis.8b00108. 29962203

[ref24] Bredin J.; Saint N.; Mallea M.; De E.; Molle G.; Pages J. M.; Simonet V. Alteration of Pore Properties of Escherichia Coli OmpF Induced by Mutation of Key Residues in Anti-loop 3 Region. Biochem. J. 2002, 363, 521–528. 10.1042/bj3630521. 11964152 PMC1222504

[ref25] Im W.; Roux B. Ion Permeation and Selectivity of OmpF Porin: A Theoretical Study Based on Molecular Dynamics, Brownian Dynamics, and Continuum Electrodiffusion Theory. J. Mol. Biol. 2002, 322, 851–869. 10.1016/S0022-2836(02)00778-7. 12270719

[ref26] Dhakshnamoorthy B.; Raychaudhury S.; Blachowicz L.; Roux B. Cation-selective Pathway of OmpF Porin Revealed by Anomalous X-ray Diffraction. J. Mol. Biol. 2010, 396, 293–300. 10.1016/j.jmb.2009.11.042. 19932117 PMC3584447

[ref27] Mahendran K. R.; Hajjar E.; Mach T.; Lovelle M.; Kumar A.; Sousa I.; Spiga E.; Weingart H.; Gameiro P.; Winterhalter M.; et al. Molecular Basis of Enrofloxacin Translocation through OmpF, an Outer Membrane Channel of *Escherichia Coli* - When Binding Does Not Imply Translocation. J. Phys. Chem. B 2010, 114, 5170–5179. 10.1021/jp911485k. 20349984

[ref28] Bodrenko I.; Bajaj H.; Ruggerone P.; Winterhalter M.; Ceccarelli M. Analysis of Fast Channel Blockage: Revealing Substrate Binding in the Microsecond Range. Analyst 2015, 140, 4820–4827. 10.1039/C4AN02293A. 25717496

[ref29] Bajaj H.; Acosta Gutierrez S.; Bodrenko I.; Malloci G.; Scorciapino M. A.; Winterhalter M.; Ceccarelli M. Bacterial Outer Membrane Porins As Electrostatic Nanosieves: Exploring Transport Rules of Small Polar Molecules. ACS Nano 2017, 11, 5465–5473. 10.1021/acsnano.6b08613. 28485920

[ref30] Golla V. K.; Sans-Serramitjana E.; Pothula K. R.; Benier L.; Bafna J. A.; Winterhalter M.; Kleinekathöfer U. Fosfomycin Permeation through the Outer Membrane Porin OmpF. Biophys. J. 2019, 116, 258–269. 10.1016/j.bpj.2018.12.002. 30616836 PMC6350074

[ref31] Acharya A.; Ghai I.; Piselli C.; Prajapati J. D.; Benz R.; Winterhalter M.; Kleinekathöfer U. Conformational Dynamics of Loop L3 in OmpF: Implications toward Antibiotic Translocation and Voltage Gating. J. Chem. Inf. Model. 2023, 63, 910–927. 10.1021/acs.jcim.2c01108. 36525563

[ref32] Richter M. F.; Drown B. S.; Riley A. P.; Garcia A.; Shirai T.; Svec R. L.; Hergenrother P. J. Predictive Compound Accumulation Rules Yield a Broad-Spectrum Antibiotic. Nature 2017, 545, 299–304. 10.1038/nature22308. 28489819 PMC5737020

[ref33] Parker E. N.; Drown B. S.; Geddes E. J.; Lee H. Y.; Ismail N.; Lau G. W.; Hergenrother P. J. Implementation of Permeation Rules Leads to a FabI Inhibitor with Activity against Gram-Negative Pathogens. Nat. Microbiol. 2019, 5, 67–75. 10.1038/s41564-019-0604-5. 31740764 PMC6953607

[ref34] Motika S. E.; Ulrich R. J.; Geddes E. J.; Lee H. Y.; Lau G. W.; Hergenrother P. J. Gram-Negative Antibiotic Active Through Inhibition of an Essential Riboswitch. J. Am. Chem. Soc. 2020, 142, 10856–10862. 10.1021/jacs.0c04427. 32432858 PMC7405991

[ref35] Parker E. N.; Cain B. N.; Hajian B.; Ulrich R. J.; Geddes E. J.; Barkho S.; Lee H. Y.; Williams J. D.; Raynor M.; Caridha D.; et al. An Iterative Approach Guides Discovery of the FabI Inhibitor Fabimycin, a Late-Stage Antibiotic Candidate with *In Vivo* Efficacy against Drug-Resistant Gram-Negative Infections. ACS Cent. Sci. 2022, 8, 1145–1158. 10.1021/acscentsci.2c00598. 36032774 PMC9413440

[ref36] Acharya A.; Jana K.; Kleinekathöfer U. Antibiotic Charge Profile Determines the Extent of L3 Dynamics in Ompf: An Expedited Passage for Molecules with a Positive Charge. J. Phys. Chem. B 2023, 127, 10766–10777. 10.1021/acs.jpcb.3c04557. 38064341

[ref37] Awasthi S.; Nair N. N. Exploring High Dimensional Free Energy Landscapes: Temperature Accelerated Sliced Sampling. J. Chem. Phys. 2017, 146, 094108 10.1063/1.4977704.

[ref38] Acharya A.; Prajapati J. D.; Kleinekathöfer U. Improved Sampling and Free Energy Estimates for Antibiotic Permeation through Bacterial Porins. J. Chem. Theory Comput. 2021, 17, 4564–4577. 10.1021/acs.jctc.1c00369. 34138557

[ref39] Jo S.; Lim J. B.; Klauda J. B.; Im W. CHARMM-GUI Membrane Builder for Mixed Bilayers and Its Application to Yeast Membranes. Biophys. J. 2009, 97, 50–58. 10.1016/j.bpj.2009.04.013. 19580743 PMC2711372

[ref40] Wu E. L.; Cheng X.; Jo S.; Rui H.; Song K. C.; Dávila-Contreras E. M.; Qi Y.; Lee J.; Monje-Galvan V.; Venable R. M.; et al. CHARMM-GUI Membrane Builder toward realistic biological membrane simulations. J. Comput. Chem. 2014, 35, 1997–2004. 10.1002/jcc.23702. 25130509 PMC4165794

[ref41] MacKerell A. D. Jr; Feig M.; Brooks C. L. Improved Treatment of the Protein Backbone in Empirical Force Fields. J. Am. Chem. Soc. 2004, 126, 698–699. 10.1021/ja036959e. 14733527

[ref42] Klauda J. B.; Venable R. M.; Freites J. A.; O’Connor J. W.; Tobias D. J.; Mondragon Ramirez C.; Vorobyov I.; MacKerell A. D. Jr; Pastor R. W. Update of the CHARMM All-Atom Additive Force Field for Lipids: Validation on Six Lipid Types. J. Phys. Chem. B 2010, 114, 7830–7843. 10.1021/jp101759q. 20496934 PMC2922408

[ref43] Darden T.; York D.; Pedersen L. Particle Mesh Ewald: An *N* log(*N*) Method for Ewald Sums in Large Systems. J. Chem. Phys. 1993, 98, 10089–10092. 10.1063/1.464397.

[ref44] Hess B. P.-L. I. N. C. S. P-LINCS: A Parallel Linear Constraint Solver for Molecular Simulation. J. Chem. Theory Comput. 2008, 4, 116–122. 10.1021/ct700200b. 26619985

[ref45] Feenstra K. A.; Hess B.; Berendsen J. C. Improving Efficiency of Large Time-scale Molecular Dynamics Simulations of Hydrogen-rich Systems. J. Comput. Chem. 1999, 20, 786–798. 10.1002/(sici)1096-987x(199906)20:8<786::aid-jcc5>3.0.co;2-b. 35619462

[ref46] Bjelkmar P.; Larsson P.; Cuendet M. A.; Hess B.; Lindahl E. Implementation of the CHARMM Force Field in GROMACS: Analysis of Protein Stability Effects from Correction Maps, Virtual Interaction Sites, and Water Models. J. Chem. Theory Comput. 2010, 6, 459–466. 10.1021/ct900549r. 26617301

[ref47] Loubet B.; Kopec W.; Khandelia H. Accelerating All-Atom MD Simulations of Lipids Using a Modified Virtual-Sites Technique. J. Chem. Theory Comput. 2014, 10, 5690–5695. 10.1021/ct500100f. 26583251

[ref48] Prajapati J. D.; Fernández Solano C. J.; Winterhalter M.; Kleinekathöfer U. Characterization of Ciprofloxacin Permeation Pathways across the Porin OmpC Using Metadynamics and a String Method. J. Chem. Theory Comput. 2017, 13, 4553–4566. 10.1021/acs.jctc.7b00467. 28816443

[ref49] Hess B.; Kutzner C.; van der Spoel D.; Lindahl E. GROMACS 4: Algorithms for Highly Efficient, Load-Balanced, and Scalable Molecular Simulation. J. Chem. Theory Comput. 2008, 4, 435–447. 10.1021/ct700301q. 26620784

[ref50] Tribello G. A.; Bonomi M.; Branduardi D.; Camilloni C.; Bussi G. PLUMED 2: New Feathers for an Old Bird. Comput. Phys. Commun. 2014, 185, 604–613. 10.1016/j.cpc.2013.09.018.

[ref51] Pettersen E. F.; Goddard T. D.; Huang C. C.; Couch G. S.; Greenblatt D. M.; Meng E. C.; Ferrin T. E. UCSF Chimera — A Visualization System for Exploratory Research and Analysis. J. Comput. Chem. 2004, 25, 1605–1612. 10.1002/jcc.20084. 15264254

[ref52] Humphrey W. F.; Dalke A.; Schulten K. VMD – Visual Molecular Dynamics. J. Mol. Graph. 1996, 14, 33–38. 10.1016/0263-7855(96)00018-5. 8744570

[ref53] Prajapati J. D.; Solano C. J. F.; Winterhalter M.; Kleinekathöfer U. Enrofloxacin Permeation Pathways across the Porin OmpC. J. Phys. Chem. B 2018, 122, 1417–1426. 10.1021/acs.jpcb.7b12568. 29307192

[ref54] Golla V. K.; Prajapati J. D.; Joshi M.; Kleinekathöfer U. Exploration of Free Energy Surfaces across a Membrane Channel Using Metadynamics and Umbrella Sampling. J. Chem. Theory Comput. 2020, 16, 2751–2765. 10.1021/acs.jctc.9b00992. 32167296

[ref55] Maragliano L.; Vanden-Eijnden E. A Temperature Accelerated Method for Sampling Free Energy and Determining Reaction Pathways in Rare Events Simulations. Chem. Phys. Lett. 2006, 426, 168–175. 10.1016/j.cplett.2006.05.062.

[ref56] Abrams J. B.; Tuckerman M. E. Efficient and Direct Generation of Multidimensional Free Energy Surfaces Via Adiabatic Dynamics without Coordinate Transformations. J. Phys. Chem. B 2008, 112, 15742–15757. 10.1021/jp805039u. 19367870

[ref57] Tripathi S.; Nair N. N. Temperature Accelerated Sliced Sampling to Probe Ligand Dissociation from Protein. J. Chem. Inf. Model. 2023, 63, 5182–5191. 10.1021/acs.jcim.3c00376. 37540828

[ref58] Pal A.; Pal S.; Verma S.; Shiga M.; Nair N. N. Mean Force Based Temperature Accelerated Sliced Sampling: Efficient Reconstruction of High Dimensional Free Energy Landscapes. J. Comput. Chem. 2021, 42, 1996–2003. 10.1002/jcc.26727. 34398461

[ref59] Hub J. S.; De Groot B. L.; Van Der Spoel D. g_wham—A Free Weighted Histogram Analysis Implementation Including Robust Error and Autocorrelation Estimates. J. Chem. Theory Comput. 2010, 6, 3713–3720. 10.1021/ct100494z.

[ref60] Maragliano L.; Fischer A.; Vanden-Eijnden E.; Ciccotti G. String Method in Collective Variables: Minimum Free Energy Paths and Isocommittor Surfaces. J. Chem. Phys. 2006, 125, 024106 10.1063/1.2212942. 16848576

[ref61] Acharya A.; Prajapati J. D.; Kleinekathöfer U. Atomistic Simulation of Molecules Interacting with Biological Nanopores: From Current Understanding to Future Directions. J. Phys. Chem. B 2022, 126, 3995–4008. 10.1021/acs.jpcb.2c01173. 35616602

[ref62] Danelon C.; Suenaga A.; Winterhalter M.; Yamato I. Molecular Origin of the Cation Selectivity in OmpF Porin: Single Channel Conductances Vs. Free Energy Calculation. Biophys. Chem. 2003, 104, 591–603. 10.1016/S0301-4622(03)00062-0. 12914905

[ref63] Hajjar E.; Mahendran K. R.; Kumar A.; Bessonov A.; Petrescu M.; Weingart H.; Ruggerone P.; Winterhalter M.; Ceccarelli M. Bridging Timescales and Length Scales: From Macroscopic Flux to the Molecular Mechanism of Antibiotic Diffusion through Porins. Biophys. J. 2010, 98, 569–575. 10.1016/j.bpj.2009.10.045. 20159153 PMC2820638

[ref64] Jeanteur D.; Schirmer T.; Fourel D.; Simonet V.; Rummel G.; Widmer C.; Rosenbusch J. P.; Pattus F.; Pages J. M. Structural and Functional Alterations of a Colicin-resistant Mutant of OmpF Porin from Escherichia Coli. Proc. Natl. Acad. Sci. U.S.A. 1994, 91, 10675–10679. 10.1073/pnas.91.22.10675. 7524100 PMC45084

[ref65] Sugawara E.; Kojima S.; Nikaido H. Klebsiella pneumoniae Major Porins OmpK35 and OmpK36 Allow More Efficient Diffusion of β-Lactams than Their Escherichia coli Homologs OmpF and OmpC. J. Bacteriol. 2016, 198, 3200–3208. 10.1128/JB.00590-16. 27645385 PMC5105900

[ref66] Golla V. K.; Prajapati J. D.; Kleinekathöfer U. Millisecond-Long Simulations of Antibiotics Transport through Outer Membrane Channels. J. Chem. Theory Comput. 2021, 17, 549–559. 10.1021/acs.jctc.0c01088. 33378186

[ref67] Haloi N.; Vasan A. K.; Geddes E. J.; Prasanna A.; Wen P.-C.; Metcalf W. W.; Hergenrother P. J.; Tajkhorshid E. Rationalizing the Generation of Broad Spectrum Antibiotics with the Addition of a Positive Charge. Chem. Sci. 2021, 12, 15028–15044. 10.1039/D1SC04445A. 34909143 PMC8612397

[ref68] Lapierre J.; Hub J. S. Converging PMF Calculations of Antibiotic Permeation across an Outer Membrane Porin with Subkilocalorie Per Mole Accuracy. J. Chem. Inf. Model. 2023, 63, 5319–5330. 10.1021/acs.jcim.3c00880. 37560945

[ref69] Vasan A. K.; Haloi N.; Ulrich R. J.; Metcalf M. E.; Wen P.-C.; Metcalf W. W.; Hergenrother P. J.; Shukla D.; Tajkhorshid E. Role of Internal Loop Dynamics in Antibiotic Permeability of Outer Membrane Porins. Proc. Natl. Acad. Sci. U.S.A. 2022, 119, e2117009119 10.1073/pnas.2117009119. 35193963 PMC8872756

[ref70] Shearer J.; Jefferies D.; Khalid S. Outer Membrane Proteins OmpA, FhuA, OmpF, EstA, BtuB, and OmpX Have Unique Lipopolysaccharide Fingerprints. J. Chem. Theory Comput. 2019, 15, 2608–2619. 10.1021/acs.jctc.8b01059. 30848905

[ref71] Lee J.; Patel D. S.; Kucharska I.; Tamm L. K.; Im W. Refinement of OprH-LPS Interactions by Molecular Simulations. Biophys. J. 2017, 112, 346–355. 10.1016/j.bpj.2016.12.006. 28122220 PMC5266143

[ref72] Lee J.; Pothula K. R.; Kleinekathöfer U.; Im W. Simulation Study of OccK5 Functional Properties in *Pseudomonas aeruginosa* Outer Membranes. J. Phys. Chem. B 2018, 122, 8185–8192. 10.1021/acs.jpcb.8b07109. 30075620

[ref73] Samsudin F.; Khalid S. Movement of Arginine through OprD: The Energetics of Permeation and the Role of Lipopolysaccharide in Directing Arginine to the Protein. J. Phys. Chem. B 2019, 123, 2824–2832. 10.1021/acs.jpcb.9b00063. 30839215

[ref74] Kesireddy A.; Pothula K. R.; Lee J.; Patel D. S.; Pathania M.; van den Berg B.; Im W.; Kleinekathöfer U. Modeling of Specific Lipopolysaccharide Binding Sites on a Gram-Negative Porin. J. Phys. Chem. B 2019, 123, 5700–5708. 10.1021/acs.jpcb.9b03669. 31260306

[ref75] Ceccarelli M.; Milenkovic S.; Bodrenko I. V. The Effect of Lipopolysaccharides on the Electrostatic Properties of Gram-Negative General Porins from Enterobacteriaceae. ChemPhysChem 2024, 25, e202400147 10.1002/cphc.202400147. 38625051

[ref76] Fajardo-Lubián A.; Ben Zakour N. L.; Agyekum A.; Qi Q.; Iredell J. R. Host Adaptation and Convergent Evolution Increases Antibiotic Resistance without Loss of Virulence in a Major Human Pathogen. PLoS Pathog. 2019, 15, e1007218 10.1371/journal.ppat.1007218. 30875398 PMC6436753

