# Supplementary material for: Molecular Mechanism of Ciprofloxacin Translocation Through the Major Diffusion Channels of the ESKAPE Pathogens Klebsiella pneumoniae and Enterobacter cloacae
Source: J Phys Chem B. 2024 Aug 24;128(35):8376–87. doi: 10.1021/acs.jpcb.4c03327 (PMC11382274; doi:10.1021/acs.jpcb.4c03327)
Supplement: Supplementary file 1 — jp4c03327_si_001.pdf [file jp4c03327_si_001.pdf]

Supporting Information: Molecular Mechanism of Ciprofloxacin Translocation through the Major Diffusion Channel of the ESKAPE Pathogens Klebsiella pneumoniae and Enterobacter cloacae

Abhishek Acharya, Pratik Kumar Behera, and Ulrich Kleinekathöfer*

School of Sciences, Constructor University, Campus Ring 1, 28759 Bremen, Germany

E-mail: ukleinekathoefer@constructor.university

Protonation states for the titrable residues of the homologs.

Previous studies on OmpF have shown that the choice of the protonation states of the titrable residues of the channel can significantly affect the dynamical fluctuations in the central L3 loop. In particular, the residues E296 and D312 appear to form a hydrogen bond network that stabilizes the L3 tip. Early investigations by Varma et al. have suggested using E296 residue as neutral to be an appropriate choice for simulations.[1] Subsequent studies have used this protonation state for simulations of OmpF,[2–4] although some studies also have employed a setup in which D312 is kept protonated in addition to E296.[5,6] Based on additional simulations of alternative protonation states, we previously showed that the protonation state suggested by Varma et al. produces the most stable L3 loop configuration consistent with the crystal structure.[4]

OmpE35

For the OmpE35, a structural superposition with OmpF (see Figure 2 in the main text) suggests that both E296 and D312 are conserved. In OmpE35 they are termed D285 and D301, respectively. Based on the structural similarity with OmpF, keeping D285 protonated and D301 charged seems justified. However, we performed protonation state calculations on OmpE35 as well using the tool Karlsberg+.[7] To this end, we set the dielectric constant of the solvent to 78.5. For the dielectric constant of the protein three different values of 4, 10, and 20 were tested, while the pH value was set to 7.0. For residue D285 we obtained estimated protonation probabilities of 1.0, 0.98, and 0.69 for protein dielectric constants of 4, 10, and 20, respectively. In the case of residue D301, we obtained probabilities of 0.13, 0.03, and 0.16, respectively. These calculations suggest that the choice of keeping D285 protonated is at least reasonable. The same protonation state was also used in a previously reported simulation study on OmpE35.[2]

OmpK35

For OmpK35, the situation is a bit more complicated. Firstly, the pore shows larger differences in the sequence when compared to OmpF, which translates to a significant difference in the titrable residues, especially in and around the constriction region as shown in Figure S1. In particular, the two residues stabilizing the L3 tip in OmpF (E296 and D312) and OmpE35 (D285 and E301) are replaced by a single residue E290 that directly interacts with the backbone of the L3 tip. The residue E290 is expected charged so that its carbonyl oxygen can act as a proton acceptor for the participating backbone amino group on the L3 tip. Apart from this, we observe two sites where there is a pair of acidic residues (E110/E20 and E102/E247) in close proximity which appears to be involved in a hydrogen bond. Due to the observed side chain configurations at these sites, one residue in each pair is likely to be protonated. To find the protonation states of these two residues, we performed calculations using Karlsberg+ employing the same setting as mentioned for OmpE35. The protonation probability of the residues E290, E110, E20, E102, and E247 using dielectric constants for the protein of 4, 10, and 20 are tabulated in Table S2. As expected, E290 is estimated to be stable in a charged state. However, for both pairs E110/E20 and E102/E247 we find a similar trend in the protonation probabilities for all values of the protein dielectric constant, that is, a decrease in the protonation state as the internal dielectric constant increases from 4 to 20. However, more importantly for both the pairs, the two residues show a significant probability of being protonated regardless of the choice of internal dielectric. In solution, it is likely that for each pair only one of the partners is protonated and the other one deprotonated, enabling the formation of a stable hydrogen bond. Thus, the two possibilities (E110 acceptor/E20 donor and E110 donor/E20 acceptor for the E110/E20 pair) are assumed to be in equilibrium, while in simulations we need to choose one of the states for modelling a stable hydrogen bond. To make a choice, we also performed calculations with the H++ server.[8] These calculations predicted a charged state for residue E290. For the pair E110/E20, the calculations predicted E110 to be protonated, while for the pair E102/E247 the residue E102

was predicted to be protonated. Based on these results, we kept E110 and E102 protonated in the OmpK35 simulations.

Table S1: Simulations systems used in the present study.

System	Atoms	Dimensions (nm × nm × nm)	Lipids	Counterions
OmpE35-CIP	135689	11.75×11.75×9.0	330	39 K^+ ions
OmpK35-CIP	138804	11.66×11.66×9.6	330	33 K_+ ions

Table S2: Protonation probabilities estimated for different titrable residue in OmpK35. The first three columns show the protonation probabilities estimated using Karlsberg+.[7] The last column lists the protonation states predicted using the H++ server.[8]

Residue	P_{H+} (4)	P_{H+} (10)	P_{H+} (20)	H++ Prediction
E290	0.0	0.0	0.0	Charged
E110	0.99	0.49	0.39	Protonated
E20	0.99	0.64	0.60	Charged
E102	1.0	0.99	0.76	Protonated
E247	1.0	0.99	0.42	Charged

Table S3: Collective variables included in the sampling scheme for the TASS simulations with the associated extended Lagrangian parameters.

**CV Type**	**CV definition**	**TAMD parameters**
Solute Translation	COM distance projections on x, y and z axes	$\kappa = 5 \times 10^5$kJ mol^{-1}nm^2<br>$\tau = 0.50$ ps
Solute Rotation	Interatomic vector projections on x, y and z axes	$\kappa = 5 \times 10^5$kJ mol^{-1}nm^2<br>$\tau = 0.40$ ps
Solute-water Interaction	Coordination Number	$\kappa = 10^4$kJ mol^{-1}nm^2<br>$\tau = 0.40$ ps
CIP-lysine Interactions	Distance	$\kappa = 10^5$kJ mol^{-1}nm^2<br>$\tau = 0.60$ ps

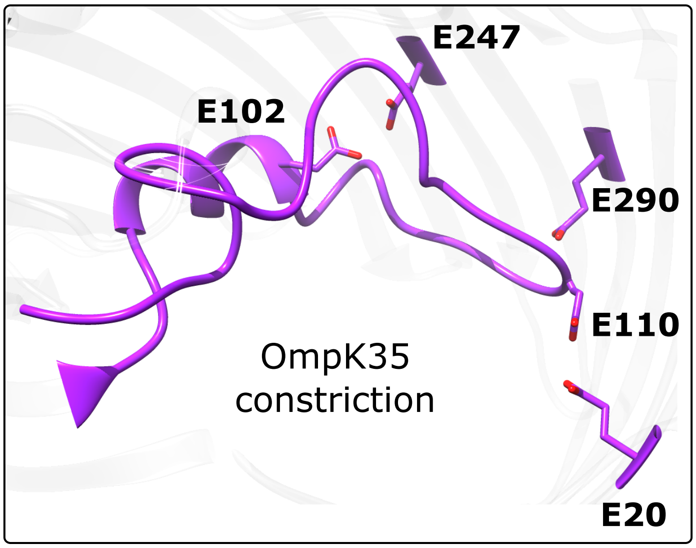

Figure S1: Titrable sites in OmpK35 considered for protonation state calculations. The other titrable residues of the channel were kept in their standard states at pH 7.0 for the simulations.

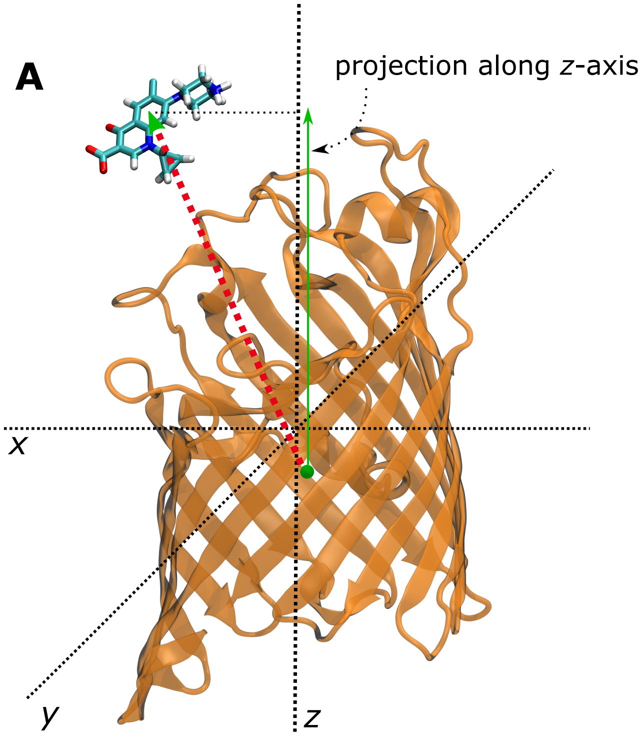

Figure S2: (A) The translocation of the antibiotic molecule through the pore is described by the collective variable z, which is defined as the projection of the distance between the center of mass of the antibiotic and the channel along the z axis. By construction, the z axis is along the pore axis. Similarly, the projection along the x and y axis was also used to bias the lateral translation of the antibiotic within the channel. (B) The rigid body rotation of the antibiotic is described by the projection of two intramolecular vectors, r_{ij}, displayed using a red arrow, and r_{kl}, shown using a blue arrow, onto the system axes. For instance, the collective variable z_{ij} represents the projection of the internal vector r_{ij} onto the z axis. See the Methods section for details.

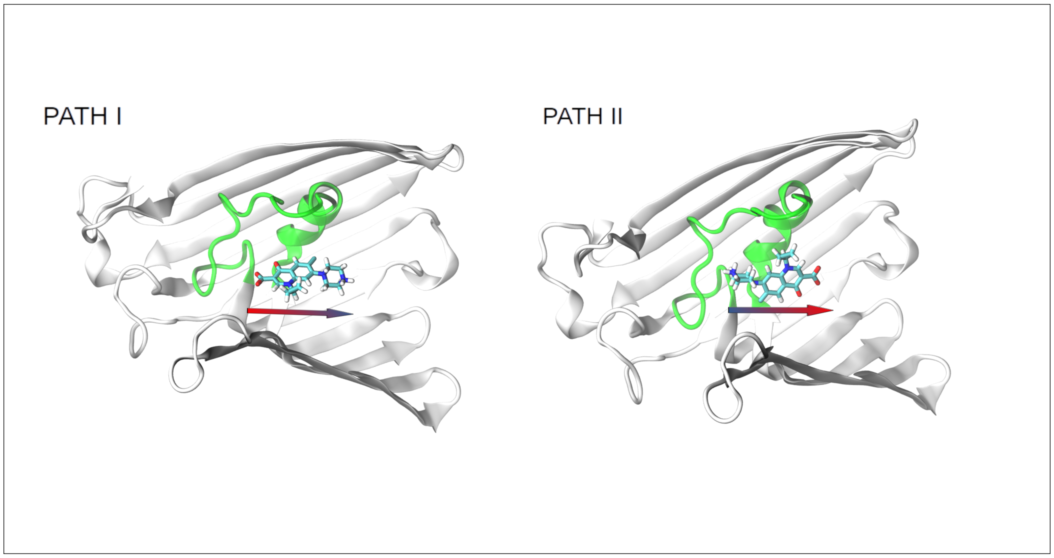

Figure S3: Ciprofloxacin can have two opposite orientations in the narrow constriction zone of the channel. Orientation I (left) with the amine group of the piperzine ring ahead as it crosses the constriction region. Orientation II (right) has the carboxylate group ahead during the passage.

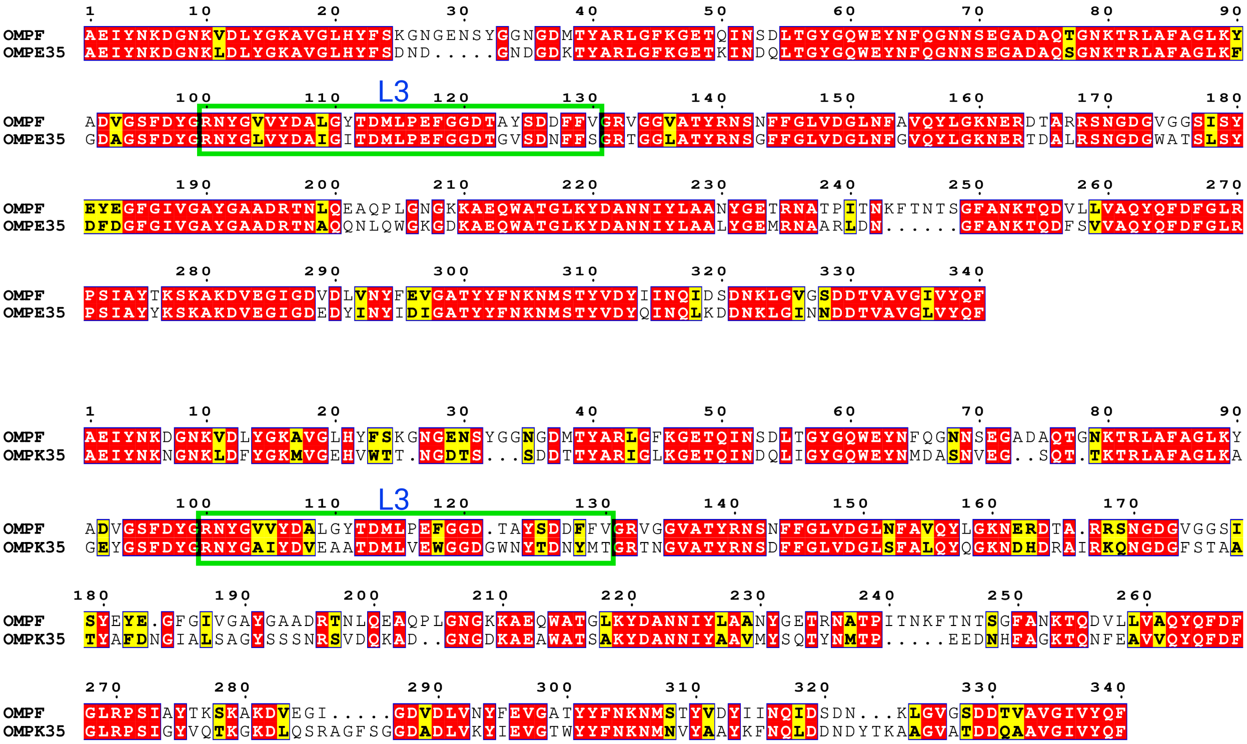

Figure S4: Pairwise sequence alignment of OmpF with OmpE35 and OmpK35.

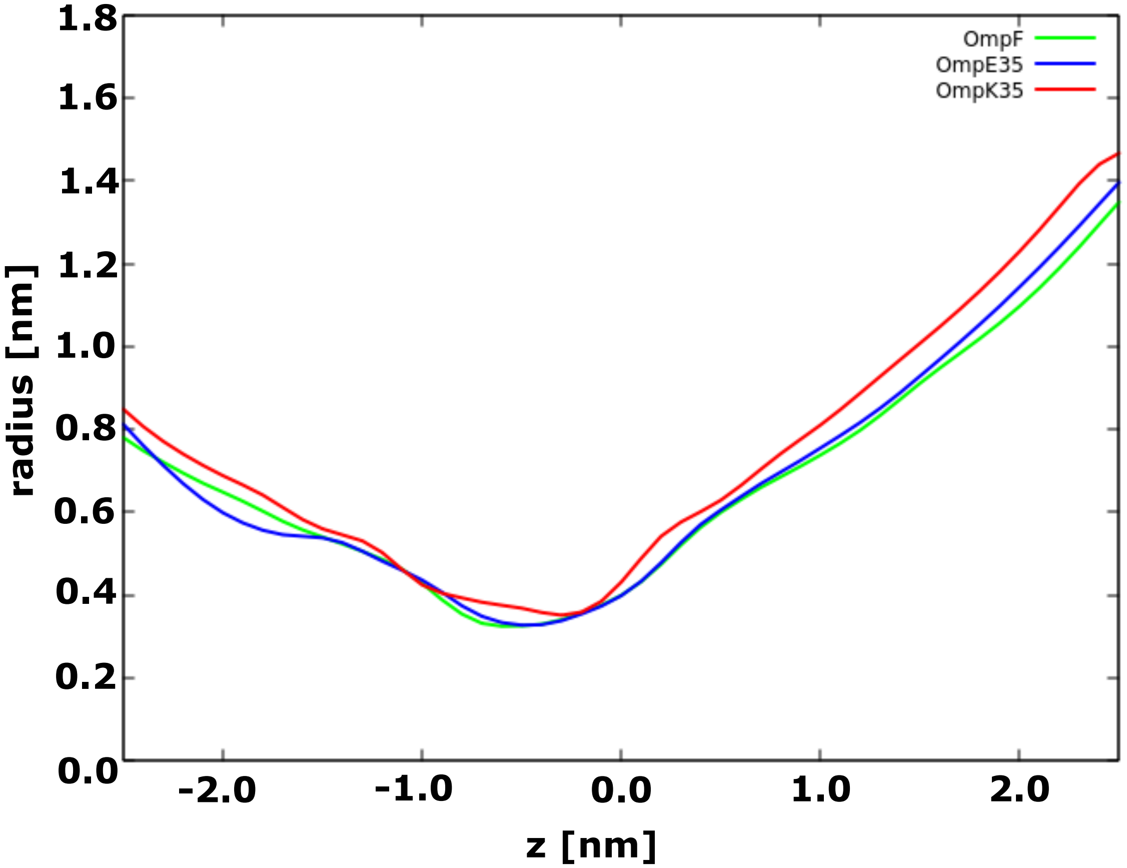

Figure S5: Profile of the channel radius along the pore axis z. The calculations were performed using the HOLE software[9] on frames extracted from the unbiased simulations, and the profiles shown here represent the average profiles from simulations.

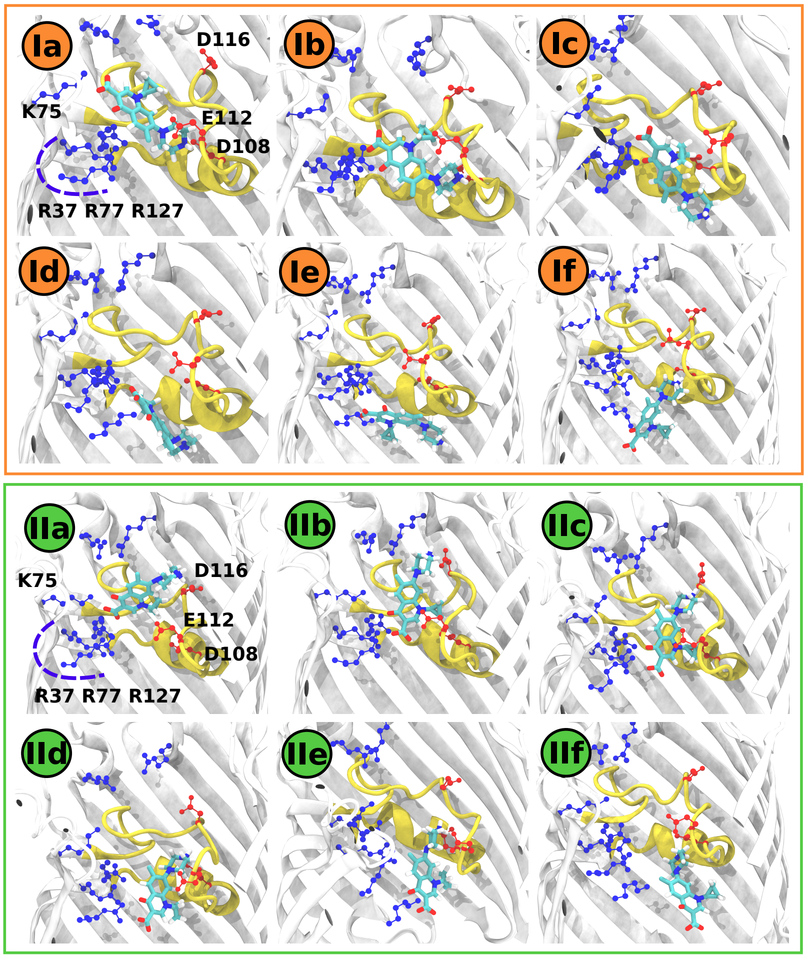

Figure S6: CIP poses in the CR of OmpE35 along the two permeation pathways. The configurations along path I and path II are marked with orange and green labels, respectively. The residues in the CR are labeled in the first panel of the respective sequence, while the L3 loop is colored in yellow. Acidic and basic residues are colored in red and blue, respectively. Note that poses Ia and IIa are the poses involved in the path-switching step depicted in Figure 6.

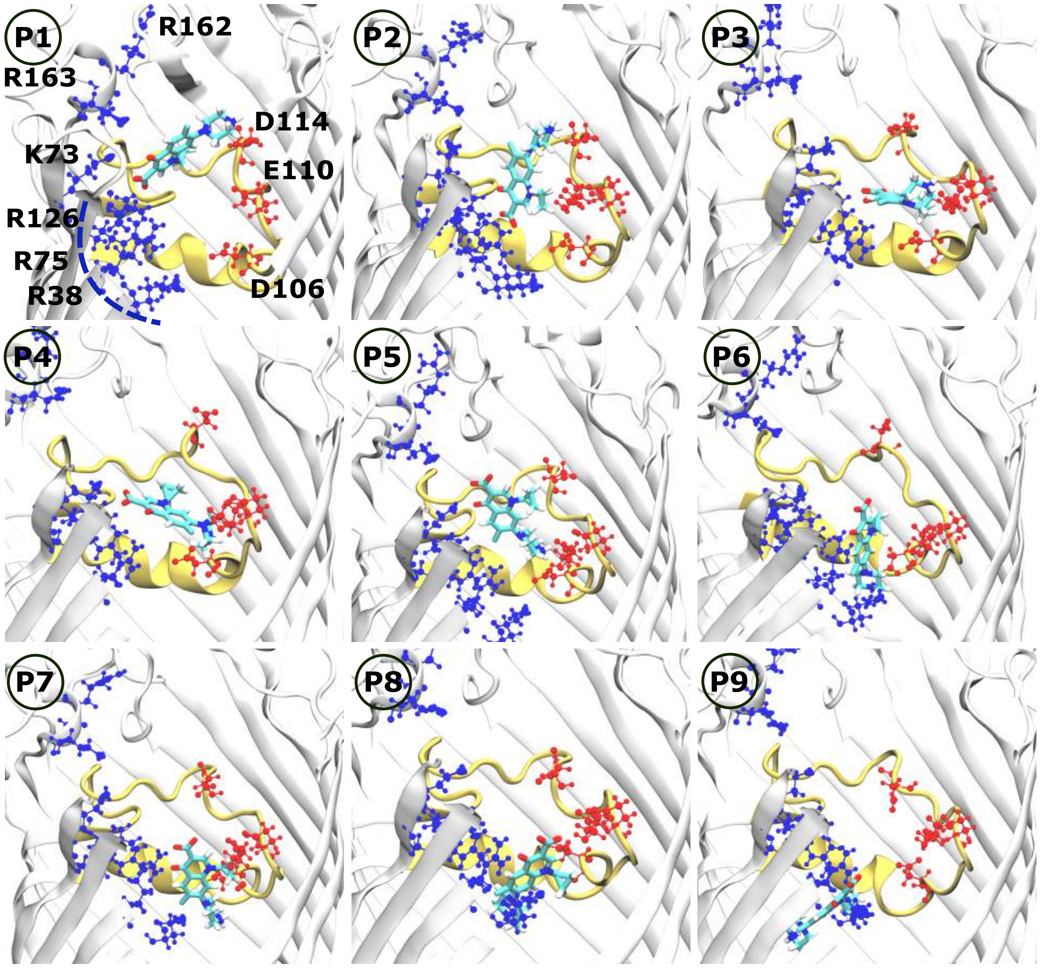

Figure S7: CIP poses in the CR of OmpK35 during translocation. The same color code as in Figure S5 has been used.

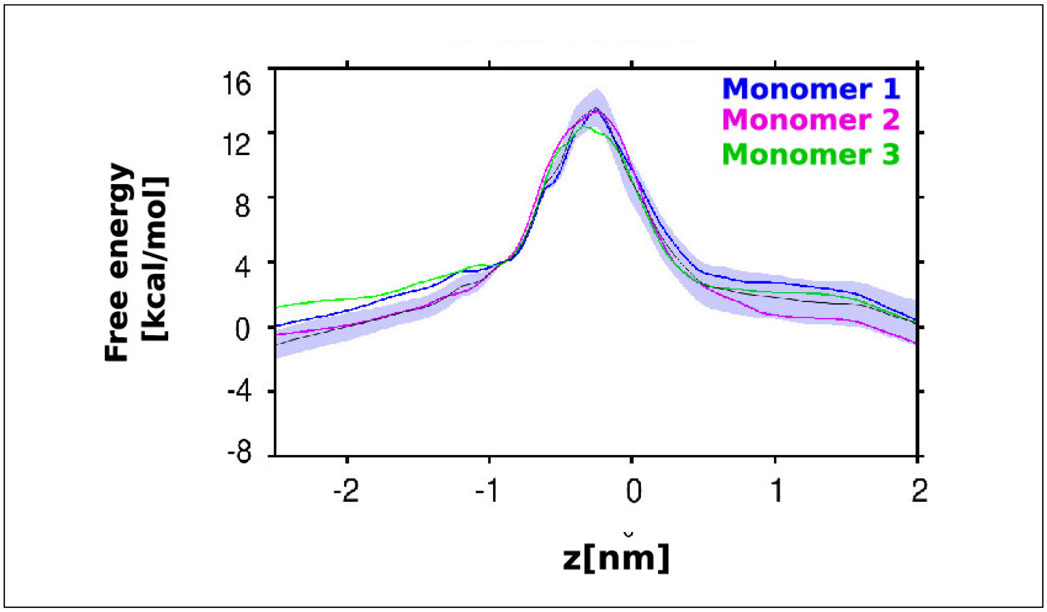

Figure S8: One dimensional free energy surface(FES) of ciprofloxacin permeation in OmpF. The figure has been adapted with permission from Acharya et al.[3] Copyright 2021 American Chemical Society.

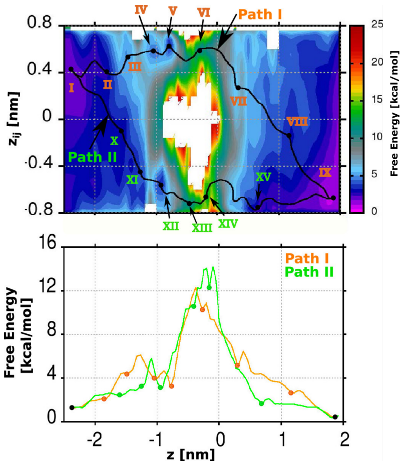

Figure S9: Two-dimensional free energy estimates for CIP permeation through OmpF and Free energy along the two pathways. The figure has been reprinted with permission from Acharya et al.[3] Copyright 2021 American Chemical Society.

References

(1) Varma, S.; Jakobsson, E. Ionization States of Residues in OmpF and Mutants: Effects of Dielectric Constant and Interactions between Residues. *Biophys. J.* **2004**, *86*, 690–704, DOI: 10.1016/s0006-3495(04)74148-x.

(2) Acosta-Gutiérrez, S.; Ferrara, L.; Pathania, M.; Masi, M.; Wang, J.; Bodrenko, I.; Zahn, M.; Winterhalter, M.; Stavenger, R. A.; Pagès, J.-M. et al. Getting Drugs into Gram-Negative Bacteria: Rational Rules for Permeation through General Porins. *ACS Infect. Dis.* **2018**, *4*, 1487–1498, DOI: 10.1021/acsinfecdis.8b00108.

(3) Acharya, A.; Prajapati, J. D.; Kleinekathöfer, U. Improved Sampling and Free Energy Estimates for Antibiotic Permeation through Bacterial Porins. *J. Chem. Theory Comput.* **2021**, *17*, 4564–4577, DOI: 10.1021/acs.jctc.1c00369.

(4) Acharya, A.; Ghai, I.; Piselli, C.; Prajapati, J. D.; Benz, R.; Winterhalter, M.; Kleinekathöfer, U. Conformational Dynamics of Loop L3 in OmpF: Implications toward Antibiotic Translocation and Voltage Gating. *J. Chem. Inf. Model.* **2023**, *63*, 910–927, DOI: 10.1021/acs.jcim.2c01108.

(5) Haloi, N.; Vasan, A. K.; Geddes, E. J.; Prasanna, A.; Wen, P.-C.; Metcalf, W. W.; Hergenrother, P. J.; Tajkhorshid, E. Rationalizing the Generation of Broad Spectrum Antibiotics with the Addition of a Positive Charge. *Chem. Sci.* **2021**, *12*, 15028–15044, DOI: 10.1039/d1sc04445a.

(6) Vasan, A. K.; Haloi, N.; Ulrich, R. J.; Metcalf, M. E.; Wen, P.-C.; Metcalf, W. W.; Hergenrother, P. J.; Shukla, D.; Tajkhorshid, E. Role of Internal Loop Dynamics in Antibiotic Permeability of Outer Membrane Porins. *Proc. Natl. Acad. Sci.* **2022**, *119*, e2117009119, DOI: 10.1073/pnas.2117009119.

(7) Rabenstein, B.; Knapp, E. W. Calculated pH-Dependent Population and Protonation

of Carbon-Monoxy-Myoglobin Conformers. *Biophys. J.* **2001**, *80*, 1141–1150, DOI: 10.1016/S0006-3495(01)76091-2.

(8) Gordon, J. C.; Myers, J. B.; Folta, T.; Shoja, V.; Heath, L. S.; Onufriev, A. H++: A Server for Estimating pKa S and Adding Missing Hydrogens to Macromolecules. *Nuc. Acids Res.* **2005**, *33*, W368–W371, DOI: 10.1093/nar/gki464.

(9) Smart, O. S.; Neduvelil, J. G.; Wang, X.; Wallace, B. A.; Sansom, M. S. P. HOLE: A Program for the Analysis of the Pore Dimensions of Ion Channel Structural Models. *J. Mol. Graph.* **1996**, *14*, 354–360, DOI: 10.1016/s0263-7855(97)00009-x.
